# Temporal Information Entropy of the Blood-Oxygenation Level-Dependent Signals Increases in the Activated Human Primary Visual Cortex

**DOI:** 10.3389/fphy.2017.00007

**Published:** 2017-02-23

**Authors:** Mauro DiNuzzo, Daniele Mascali, Marta Moraschi, Giorgia Bussu, Bruno Maraviglia, Silvia Mangia, Federico Giove

**Affiliations:** 1 Division of Glial Disease and Therapeutics, Faculty of Health and Medical Sciences, Center for Basic and Translational Neuroscience, University of Copenhagen, Copenhagen, Denmark; 2 Museo Storico della Fisica e Centro Studi e Ricerche Enrico Fermi, Rome, Italy; 3 Fondazione Santa Lucia (IRCCS), Rome, Italy; 4 Department of Radiology, Center for Magnetic Resonance Research, University of Minnesota, Minneapolis, MN, USA

**Keywords:** fMRI, BOLD signal distribution, Shannon information entropy, visual stimulation, visual system

## Abstract

Time-domain analysis of blood-oxygenation level-dependent (BOLD) signals allows the identification of clusters of voxels responding to photic stimulation in primary visual cortex (V1). However, the characterization of information encoding into temporal properties of the BOLD signals of an activated cluster is poorly investigated. Here, we used Shannon entropy to determine spatial and temporal information encoding in the BOLD signal within the most strongly activated area of the human visual cortex during a hemifield photic stimulation. We determined the distribution profile of BOLD signals during epochs at rest and under stimulation within small (19–121 voxels) clusters designed to include only voxels driven by the stimulus as highly and uniformly as possible. We found consistent and significant increases (2–4% on average) in temporal information entropy during activation in contralateral but not ipsilateral V1, which was mirrored by an expected loss of spatial information entropy. These opposite changes coexisted with increases in both spatial and temporal mutual information (i.e., dependence) in contralateral V1. Thus, we showed that the first cortical stage of visual processing is characterized by a specific spatiotemporal rearrangement of intracluster BOLD responses. Our results indicate that while in the space domain BOLD maps may be incapable of capturing the functional specialization of small neuronal populations due to relatively low spatial resolution, some information encoding may still be revealed in the temporal domain by an increase of temporal information entropy.

## INTRODUCTION

The understanding of information encoding in the brain, i.e., how the brain represents and processes information, is an important goal for neuroscience [1]. Measurement of brain activity is paramount for attempting a quantitative analysis of information encoding, a goal that can be approached with different experimental techniques that operate at different spatiotemporal scales. Accordingly, data can be obtained from a variety of cellular processes, ranging from neuronal action potentials in individual neurons to integrated neuron-glia functional interactions within large populations of cells. In particular, functional Magnetic Resonance Imaging (fMRI) measures macroscopic changes in blood-oxygenation level-dependent (BOLD) signals. As such, fMRI-BOLD signals reflect neuronal activity only indirectly through the mediation of the neuro-vascular and neuro-metabolic coupling [2].

Conventional fMRI provides spatial and temporal resolution in the order of cubic millimeters and seconds, respectively. Therefore, fMRI studies have commonly been used to extract information (e.g., activations and/or deactivations) about relatively slow processes (e.g., task-related vascular responses) taking place in tissue volumes relatively larger than the underlying functional units (e.g., cortical areas vs. columns). By using global spatiotemporal BOLD patterns, it has been recently demonstrated that fMRI can decode information about content of watched movies [3], imagery [4], and dreams [5]. Although the spectral properties of temporal autocorrelation of BOLD oscillations have been convincingly proposed as a metric for its temporal organization, these concepts were developed for the analysis of fluctuations of the resting brain, and do not directly apply to the study of localized, evoked responses [6].

Based on prior knowledge about the stimulation protocol as well as model of neural-hemodynamic response [7], analysis of fMRI data obtained during photic sensory stimulation consistently reveals clusters of activated, and to a lesser extent deactivated [8, 9], voxels in primary visual cortex (V1). These voxels identify the main effect of stimulation whereby they exhibit a common, stereotyped temporal BOLD response that conforms to the specified model and set of assumptions. Much effort has been devoted to the mapping of the spatial information processing in early visual areas [10–15]. In addition to the investigation of the topologic mapping of cortical inputs or function, few studies have investigated the behavior of voxels *within* responsive clusters to examine intrinsic information encoding in the BOLD time series.

The purpose of this work was to quantify the spatial and temporal content of information associated with the BOLD signals in the activated human primary visual cortex. We used Shannon entropy as it is a simple, univariate measure of the information content of a signal that does not require prior knowledge or comparisons with other signals. Furthermore, Shannon entropy can be easily determined by estimation of the probability distribution function and thus it has a clear statistical meaning. Stimulation-induced changes in temporal information entropy of BOLD time-series within activated sensory functional areas have been previously reported to either increase [16] or decrease [17]. Intracluster spatial information entropy has not been directly assessed. However, a decrease in spatial information entropy is suggested by several works that reported increases in spatial homogeneity (i.e., temporal correlation or concordance of BOLD time-series in adjacent voxels) within activated sensory areas both in humans [18] and in rodents [19], although the results may depend on the temporal frequency band [20] and brain region [21]. In the primary visual cortex, stable photic stimuli induce very reproducible activation patterns [22] that are compatible with a task-related decrease of spatial information entropy, albeit functionally homogeneous areas show a great degree of synchronization even at rest [23].

Here we examined the stimulation-induced changes in spatial and temporal information entropy encoded in the BOLD signal, and we assessed the stimulation-induced change in intracluster correlation between voxel time series by mutual information dependence analysis, both spatially and temporally. Our analysis provided insights into the relationship between spatial distribution and temporal synchronization of the voxels constituting a single activated cluster while they collectively respond to a relevant stimulation.

## METHODS

### Subjects

Fifteen healthy subjects (age = 25 ± 7 y.o., mean ± SD; female/male = 5/10) participated in the study. Exclusion criteria included vascular and neurological disease, history of epileptic seizure and any contraindication to MRI. All enrolled subjects were informed before they signed the consent form to take part in the study, according to the Helsinki declaration and to European Union regulations. The study was carried out in accordance with a protocol approved by the local Ethics Committee and in agreement with the institutional guidelines.

### Data Acquisition

All images were acquired on a 3T MRI Scanner (Siemens Magnetom Allegra, Erlangen Germany) equipped with a standard birdcage coil. T_1_-weighted images were acquired as anatomical reference using a Magnetization-Prepared Rapid Acquisition Gradient Echo (MPRAGE) sequence (TR/TE/TI = 2150/2.48/1000 ms, Flip Angle = 8°, voxel size = 1 × 1 × 1 mm^3^, FOV = 256 × 160 mm^2^, 176 paraxial slices). Functional images were acquired with a Gradient Echo-Echo Planar Imaging (GE-EPI) sequence (TR/TE = 2000/30 ms, Flip Angle = 80°, voxel size = 3 × 3 × 3.75 mm^3^, FOV = 192 × 192 mm^2^, 32 paraxial slices, 3 mm slice thickness with 0.75 mm slice spacing, covering the whole brain).

### Paradigm and Stimulation

The visual stimulation was generated using Cogent 2000 (Laboratory of Neurobiology, Wellcome Trust, London, UK) under Matlab 7.1 (The Mathworks Inc, Natick, Massachusetts, USA) and delivered during functional scans through a Digital Light Processing (DLP) projector. The projector was located outside the MR scanner room, and it projected the stimulus on a screen positioned on the magnet bore behind the subject, who viewed it by a mirror positioned on the head coil.

The visual stimulus (Figure S1A) consisted of a circle-shaped (3° of radius) white-black rotating (2 cycles/s) checkerboard in either right or left hemifield, at 4° of horizontal decentering with uniform gray background. The checkerboards changed rotation direction with period ranging uniformly between 1 and 3 s. The stimulus included an area of the visual field coverage of V1 that is expected to elicit uniformly strong activation within the contralateral V1 and no activation in the ipsilateral V1 [13]. The stimulation was administered in a block design, with periods of stimulation alternated with rest (full uniform gray field). Fixation point at the center of the screen was identified by a white cross rotating by 45° with a random period ranging from 1.5 to 4.5 s during the whole acquisition. The subjects were required to fix the central cross, and subject’s gaze was monitored (sampling rate 60 Hz) through an eye-tracking system (Applied Science Laboratories, model 504) equipped with remote pan/tilt optic infrared module and a camera that was custom-adapted for use in the scanner. Attention was monitored by asking the subjects to push a bottom for every rotation of the fixation cross. The fMRI stimulation paradigm (Figure S1B) consisted of four cycles, each comprising 3 epochs (either right hemifield stimulation (R), left hemifield stimulation (L), rest (r), or L,R,r) for a total of 12 epochs. Each epoch was 30 s long, resulting in a total session duration of 6 min (i.e., 180 volumes, not including 4 dummy scans before the start of the stimulation that were discarded before any processing).

### Image Processing

Data underwent standard preprocessing using SPM8 (Statistical Parametric Mapping) Matlab toolbox (Wellcome Trust Centre for Neuroimaging, London, UK; http://www.fil.ion.ucl.ac.uk/spm/software/spm8/). All functional volumes were first realigned to their mean using a two iterations approach, and then corrected for slice-timing. Both steps used the default SPM8 settings. Since this work is focused on ROI-based parameters that were calculated on each subject’s space, spatial normalization was not performed. Smoothing was not applied, in order to preserve the intrinsic interdependency between voxels.

### Assessment of the Responding Functional Areas

GLM analyses were carried out in the time-domain on a voxel-by-voxel basis by modeling the functional signal as the convolution of the default (i.e., SPM8 built-in) hemodynamic response function with the task paradigm. Prior to regression, BOLD time series were high-pass filtered with a cut o3 period of 135 s in order to remove low-frequency noise. Statistical inference was performed by means of one-sample *t*-test on the regression model. For each subject and stimulus condition the task-responding functional area was defined as the most significant cluster of GLM t-map (p-FWE < 0.0001 at cluster level), and was consistently located in contralateral visual areas, around the calcarine sulcus. The cluster was obtained by the standard clustering algorithm provided by SPM8.

### ROI Definition

First, we defined spherical ROIs of different volumes centered around the *t*-peak voxel of the most activated cluster (as defined above) of each subject. Then, we constrained the ROIs to belong entirely to the activated cluster while maintaining the desired volume. Therefore, in case the spherical ROI was not completely included in the activation cluster, the following algorithm was applied. Contiguous voxels pertaining to successive spherical surfaces (with radius increasing at 2 mm steps) and belonging to the same cluster around the activation peak were sorted for significance and progressively included into the ROI until the desired volume was reached. Although this approach produces ROIs of different shapes, it ensures the number of voxels to be the same across subjects, allowing for second-level comparisons. The nominal radius of the resulting ROIs (i.e., the radius of the sphere equivalent to the ROI) varied from 5 to 10 mm. The upper bound for ROI size (i.e., 10 mm) was limited by statistical significance. The lower bound for ROI size (i.e., 5 mm) was limited by setting the minimum number of voxels required for determination of statistical distribution to the maximum value returned by Freedman-Diaconis rule based on interquartile range (IQR) [24].

This approach is unsuited to exactly identify the cortical area whose neuronal receptive field matches the stimulus location [25], however it has the advantage of relying on the data themselves, not requiring further acquisitions spatially coregistered with the main experiment data, and proved successful in the identification of ROIs reproducibly and strongly activated by the stimulus.

### Distribution Analysis

All calculations were performed using custom MATLAB routines. Raw BOLD signals were first intensity normalized in order to remove the dependence of signal change on intrinsic voxel intensity while retaining the natural signal measure and associated dispersion [26].

The distribution profiles of the resulting values within a ROI as well as distribution differences were determined using Gaussian kernel density estimation [27]. Mean, variance, excess kurtosis and absolute skewness of the distribution were calculated using associated moments. Normality of the distribution was determined using the Anderson-Darlin test [28]. Information entropy was calculated according to the standard Shannon formulation (i.e., as statistical entropy):

$$H=-\sum_{k}p_{k}\log p_{k}$$

where *p_k_* denotes the probability for the system to be in the *k*-th state and satisfies the normalization condition:

$$N=\sum_{k=1}^{N_{k}}n_{k}\to 1=\sum_{k=1}^{N_{k}}p_{k}$$

with *n_k_* being the number of occurrences of the *k*-th state and *N_k_* being the total number of states. Based on the probability distribution function (see below), the individual probabilities *p_k_* were calculated from either the entire time-course of the BOLD signal in each individual voxel (time-domain entropy) or the values of BOLD signal in an entire ROI for each individual time point (space-domain entropy). It is noted that time-domain entropy turns out to be a function of space (i.e., it is a map), whereas space-domain entropy turns out to be a function of time (i.e., it is a time course). In particular, denoting the BOLD signal as *s*(*x*, *y*, *z*, *t*) the time-domain entropy is defined as:

$$H(x,y,z)=-\sum_{k}p_{k}(x,y,z)\log p_{k}(x,y,z)$$

where the probabilities *p_k_* are determined *across* the time variable *t* and therefore no longer depend on it, which gives:

$$p_{k}(x,y,z)=\sum_{i=1}^{N_{T}}I_{k}(t_{i})$$

where *I_k_*(·) is the indicator function of the *k*-th state and *N_T_* is the total number of time points in the fMRI data series. Similarly, the space-domain entropy is defined as:

$$H(t)=-\sum_{k}p_{k}(t)\log p_{k}(t)$$

where the probabilities *p_k_* are determined *across* the space variables, *y*, and *z* and therefore no longer depend on them, which gives:

$$p_{k}(t)=\sum_{i=1}^{N_{V}}I_{k}(x_{i},y_{i},z_{i})$$

where *N_V_* is the total number of voxels across the three spatial dimensions of a given region of space.

The probability density function was determined by the histogram method using the Freedman-Diaconis rule [24], that is:

$$N_{k}=2\frac{\mathrm{IQR}[s(x,y,z)]}{\sqrt[{3}]{N_{T}}}$$

or

$$N_{k}=2\frac{\mathrm{IQR}[s(t)]}{\sqrt[{3}]{N_{V}}}$$

for time- and space-domain, respectively.

As a measure of dependence, we used mutual information *M*_1,2_ in both space- and time-domain. Mutual information was computed as the difference between the sum of marginal entropies and the joint entropy of each pair of voxels within a ROI, according to:

$$M(x_{1},y_{1},z_{1},x_{2},y_{2},z_{2})=H(x_{1},y_{1},z_{1})+H(x_{2},y_{2},z_{2})-H(x_{1},y_{1},z_{1},x_{2},y_{2},z_{2})$$

or

$$M(t_{1},t_{2})=H(t_{1})+H(t_{2})-H(t_{1},t_{2})$$

for time- and space-domain, respectively.

Entropy values were normalized to the theoretical entropy maximum (for finite support, this equals base-2 logarithm of the number of data points). When averaging, parameters that are function of time were additionally shifted in time by 3 TR (chosen as the best shift to center the plateau of the positive BOLD response in the epoch window) to take into account the hemodynamic delay.

## RESULTS

**FIGURE 1:**
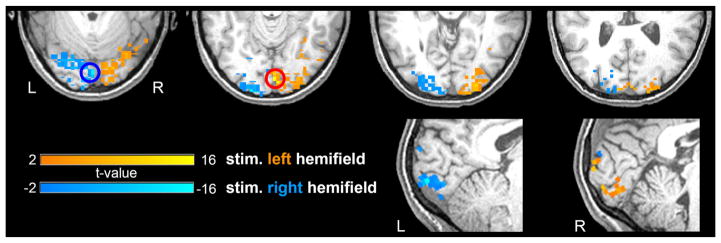
Activation patterns elicited by the visual stimulation Combined activations for a representative subject. Hot and cold colormaps represent voxel-wise response to stimulation in the left or right visual hemifield, respectively. The most activated voxel for each condition, center of the ROI definition procedure, is highlighted by a circle. The parametric t-map is overlaid on the MPRAGE scan of the same subject.

As expected, statistical maps obtained with GLM revealed task-related activations in visual areas contralateral to the hemifield visual stimulation, putatively within V1 and peaking around the calcarine sulcus (Figure 1). To examine the effect of spatial extension of the activated area on the collective behavior of its constituting voxels, we used 6 ROIs of nominal radius from a minimum of 5 mm to a maximum of 10 mm, extracted as described in the ROI definition subsection within Methods (Table 1).

**FIGURE 2:**
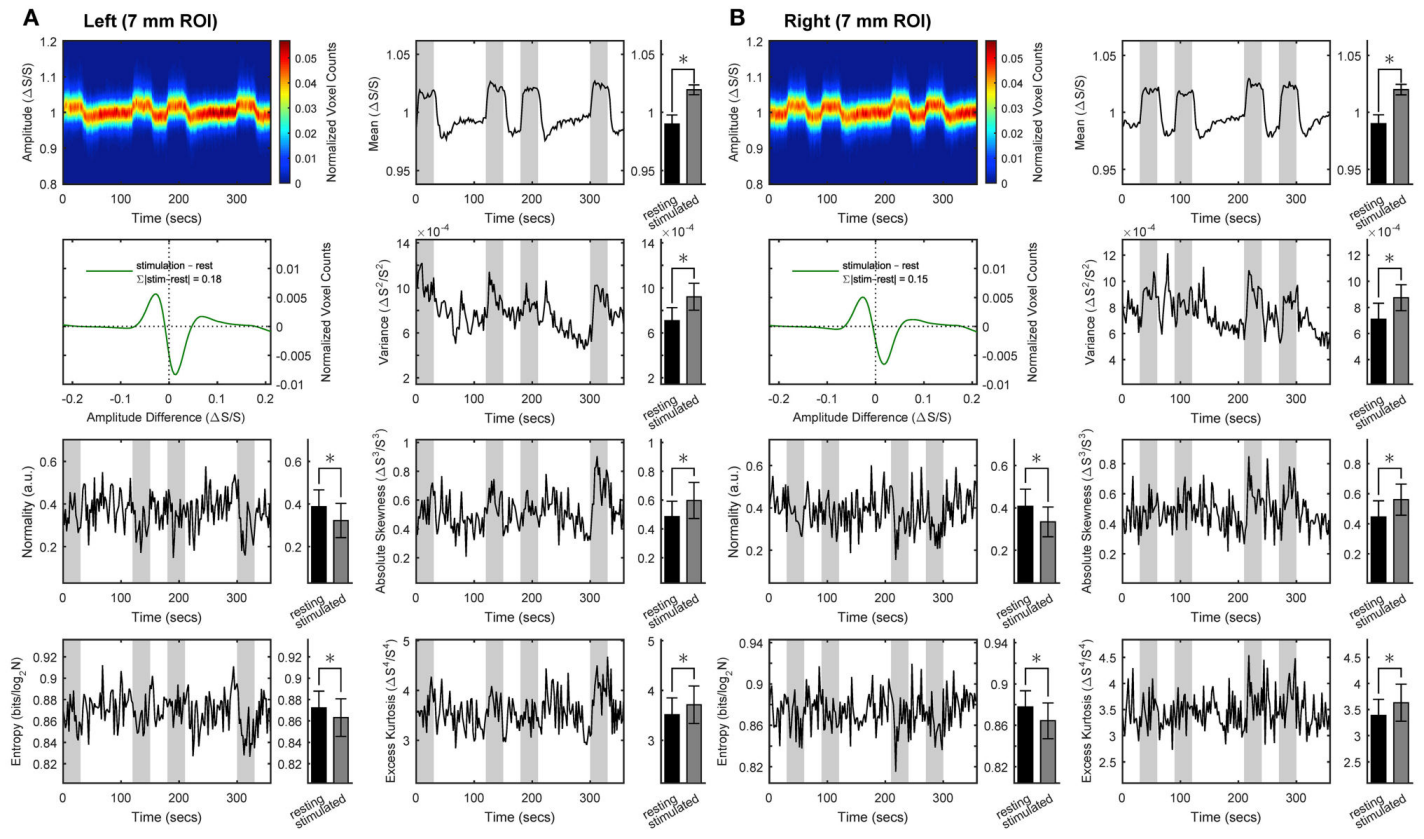
Space-domain analysis of BOLD distribution in activated primary visual cortex Group results for the ROIs (here, representatively of radius 7 mm) located in left **(A)** or right **(B)** primary visual cortex. Left in each panel (from top to bottom): intersubject average time-courses of distribution profile of the within-ROI BOLD values, z-transformed distribution of BOLD values difference between stimulus and rest, and intersubject average time-courses of distribution normality and information entropy. Right in each panel (from top to bottom): intersubject average time-courses of distribution mean, variance, absolute skewness and excess kurtosis. Epochs of stimulation are marked with gray-shaded areas. All distribution profiles and time-courses were determined for each subject individually and then averaged across subject. Average values (bar plots on the right of each time-resolved plot) are expressed as mean ± sd (*n* = 15) and statistical threshold is set to *p < 0.001.

To assess whether rest and stimulus conditions could be characterized in terms of the organization of within-ROI voxels, we performed space-domain distribution analysis of BOLD signals. Figure 2 shows the results obtained for the medium ROI (7 mm; see Figures S2–S6 for the other ROI sizes). We found that spatial BOLD distribution profile (i.e., independent of mean and variance) is significantly altered in the transition from rest to stimulus. Specifically, within the activated ROI (bilaterally) we found small but statistically significant decreases in spatial information entropy as well as in distribution normality during stimulation compared with rest. These decreases were accompanied by increases in absolute skewness and excess kurtosis. The latter two features entails that the distribution of BOLD values within the ROI is more peaked and asymmetric during stimulus than at rest, which was confirmed by calculating the BOLD z-transformed distribution differences. These findings indicate that while processing the stimulus a fraction of responding voxels span a narrower range of intensities compared with rest. As indicated by the sum of the absolute distribution differences, the rearranged voxels represented 18% (left ROI) or 15% (right ROI) of the total. At the same time, the whole distribution exhibits a broader profile and a corresponding increase in variance, as supported by Shannon inequality for non-Gaussian distributions (see for example [29]). Overall, during stimulation the space-domain distribution profile changed substantially relative to rest, as evidenced by the increase in mean, variance, skewness and kurtosis as well as the decrease in normality and entropy. Note that quantitatively only mean and variance rely on the actual values of the BOLD signals, whereas the other quantities only depend on the profile of the underlying distribution.

**FIGURE 3:**
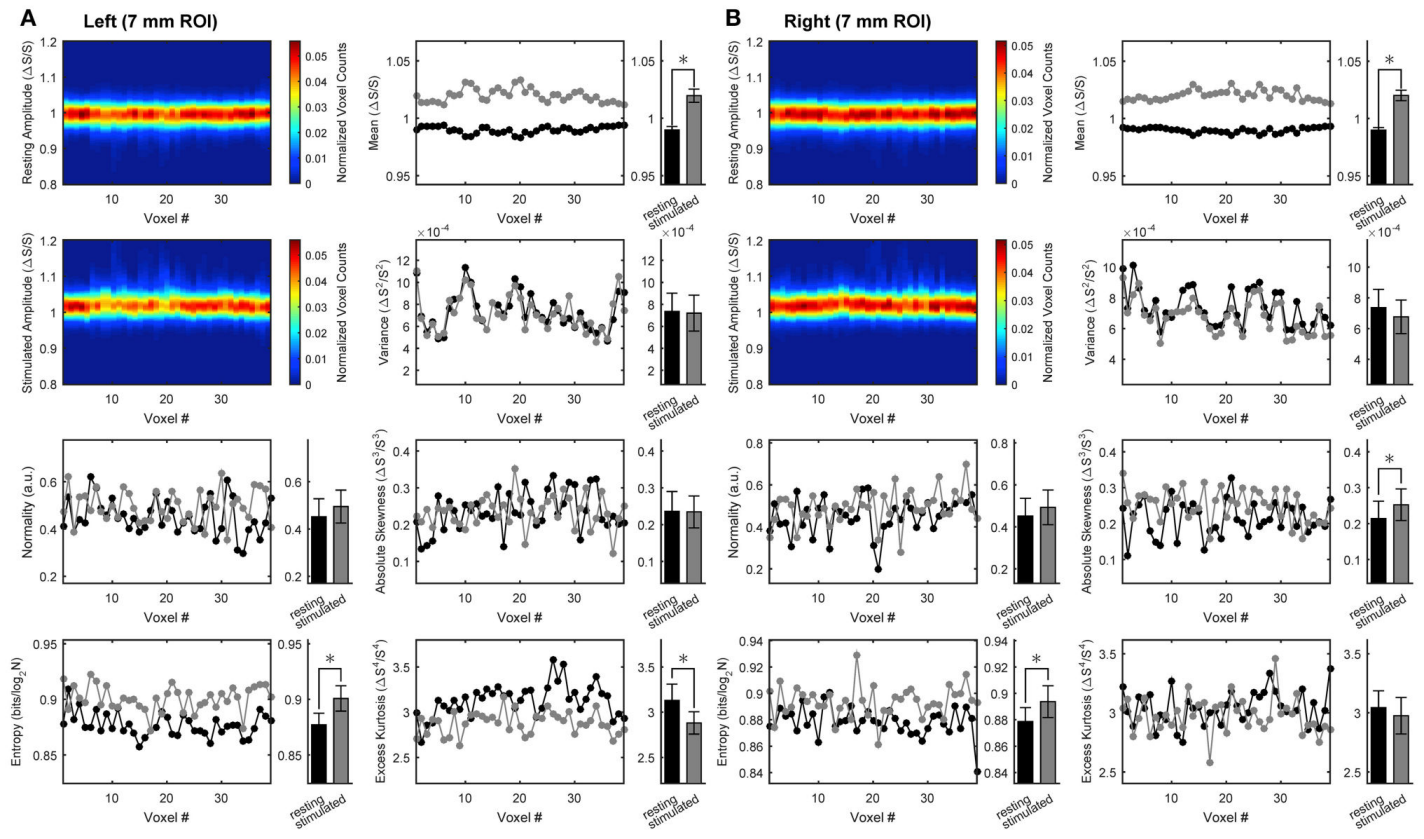
Time-domain analysis of BOLD distribution in activated primary visual cortex Group results for the ROIs (here, representatively of radius 7 mm) located in left **(A)** or right **(B)** primary visual cortex. Left in each panel (from top to bottom): intersubject average arrangements of the distribution profile of the within-ROI BOLD values at rest as well as during stimulation, and intersubject averages of rest vs. stimulus distribution normality and statistical entropy. Right in each panel (from top to bottom): intersubject averages of within-ROI time-courses of rest vs. stimulus distribution mean, variance, absolute skewness and excess kurtosis. All distribution profiles and voxel arrangements were determined for each subject individually and then averaged across subject. Average values (bar plots on the right of each space-resolved plot) are expressed as mean ± sd (n **=** 15) and statistical threshold is set to *p < 0.001.

To assess stimulation-induced changes in temporal variables, we performed time-domain distribution analysis of BOLD signals. Figure 3 shows the results obtained for the same medium ROI (7 mm; see Figures S7–S11 for the other ROI sizes). During stimulation, the time-domain distribution profile changed very little relative to rest, excluding the straightforward translation of the mean toward higher values. Nonetheless, temporal information entropy increased significantly during stimulation compared with rest. The effect was consistently observed in both hemispheres under stimulation. Indeed, the significant changes in other distribution-related quantities (here excess kurtosis in the left ROI and absolute skewness in the right ROI) are not consistently bilateral across all ROIs (see also Figures S7–S11).

The fact that the change in temporal information entropy was the only change that was consistently observed contralateral to both hemifield stimuli indicates that the entropy measure captures some features of the BOLD temporal dynamics that escape other distribution-based quantities. The above changes in spatial and temporal information entropy, as well as the variations of the other quantities associated with the BOLD distribution (except mean and variance), were found to be independent of ROI size (Table 2). Notably, conditions with subject at rest were indistinguishable from conditions with ipsilateral stimulation, hence both conditions were pooled together (e.g., in all bar plots).

**FIGURE 4:**
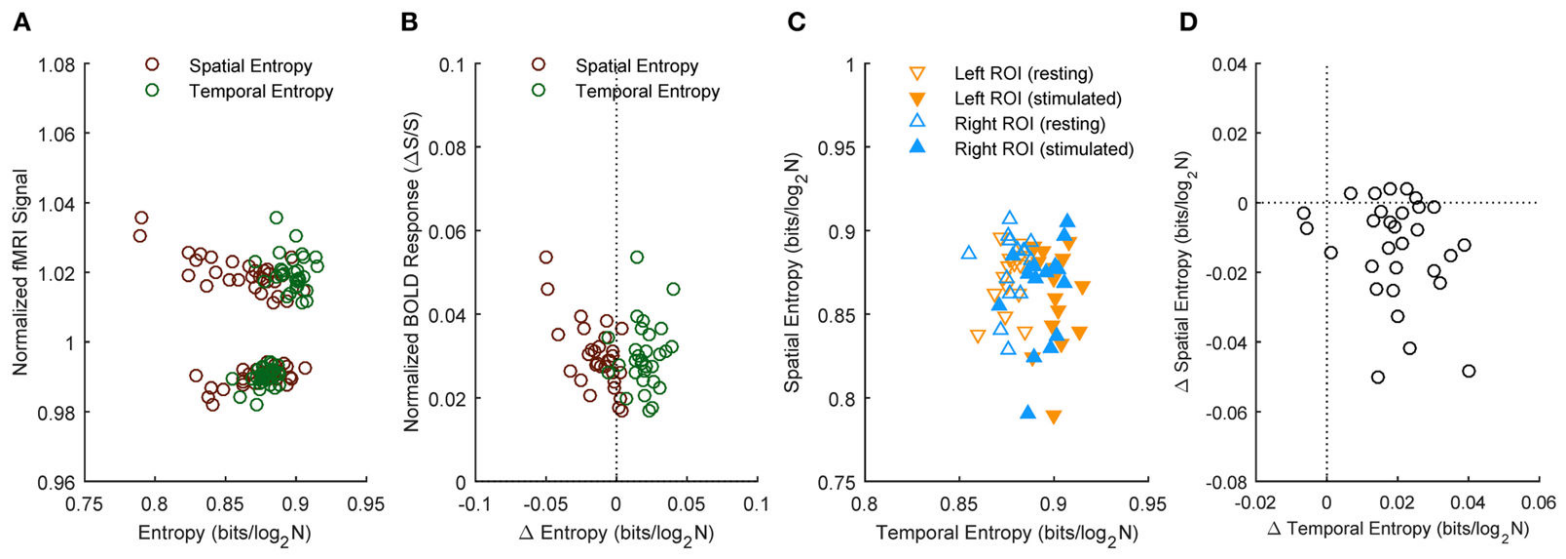
Correlation between spatial and temporal information entropy in activated primary visual cortex Subject results for the ROIs (here, representatively of radius 7 mm) located in left or right primary visual cortex. Information entropy and normalized fMRI signal **(A)** as well as their stimulation-induced changes **(B)** reveal no correlation. Likewise, there is no correlation between temporal and spatial entropy **(C)**, or between their changes during stimulation **(D)**.

To examine possible relations between spatial and temporal information, we performed correlation analysis. Figure 4 shows the results obtained for the medium ROI (7 mm; see Figures S12–S16 for the other ROI sizes). Except for the clear-cut relation between rest and stimulated values of spatial or temporal information entropy and the corresponding fMRI signals (Figure 4A), we found no correlation between the stimulation-induced changes of information and BOLD response (Figure 4B, *p* > 0.46). Similarly, we observed no significant correlations between absolute values of spatial and temporal information entropy (Figure 4C, *p* > 0.19) and between their changes (Figure 4D, *p* > 0.18). The lack of correlation between the stimulus-related increase of temporal entropy and the amplitude of the BOLD response suggests that the reported encoding of information in BOLD temporal oscillations is not biased by contrast to noise ratio. The lack of correlation between changes in temporal and spatial entropy indicates that the stimulus-related increase of temporal entropy is independent of change of spatial encoding of information within the activated area.

**FIGURE 5:**
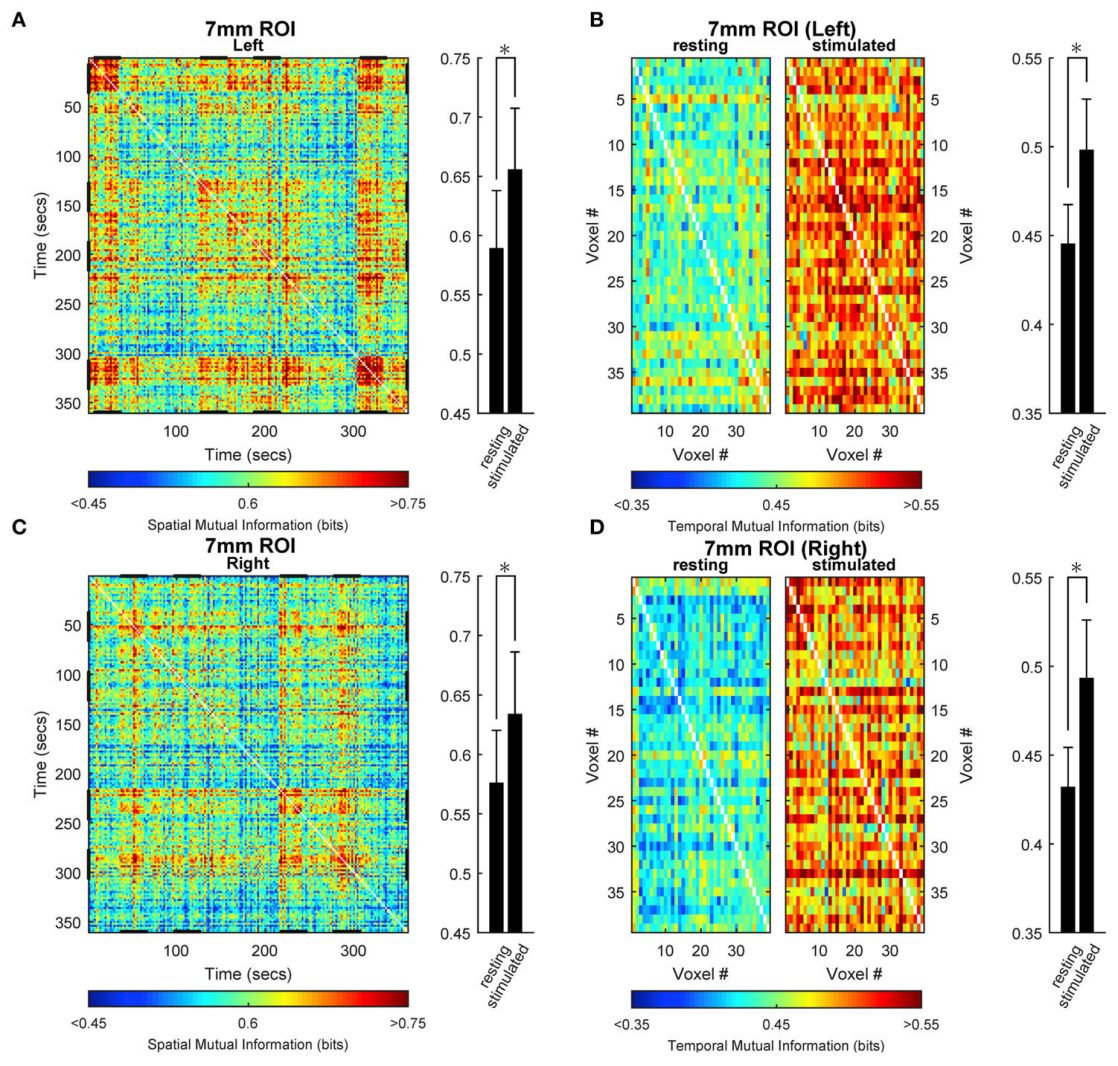
Space- and time-domain mutual information analysis in activated primary visual cortex Group results for the ROIs (here, representatively of radius 7 mm) located in left **(A,B)** or right **(C,D)** primary visual cortex. **(A,C)** Spatial mutual information. **(B,D)** Temporal mutual information for rest (right in each panel) and stimulus (left in each panel) conditions. Spatial mutual information (single-subject) has been computed from the arrangements of voxels in each time instant. Temporal mutual information (single-subject) has been computed from the voxel time courses in each epoch of rest and stimulation independently and subsequently averaged. The individual matrices so obtained for each subject have been then averaged across all subjects to obtain the present images. Note that the difference between values at rest and under stimulation for spatial mutual information have been obtained by averaging the matrices in the relevant rest and stimulation epochs (different for each side, stimulation epochs are highlighted by a bolder tract in x and y axes, **A,C**). During stimulation, the degree of general dependence as measured by mutual information, increases relative to rest both spatially and temporally. Average values (bar plots on the right of each time- or space-resolved plot) are expressed as mean ± sd (*n* = 15) and statistical threshold is set to *p < 0.001.

To examine how the opposite stimulation-induced changes in spatial and temporal information entropy are related to the degree of spatial and temporal dependence within the activated ROI, we determined the mutual information in both space- and time-domain. Figure 5 shows the results obtained for the medium ROI (7 mm; see Figures S17–S21 for the other ROI sizes). Spatial (Figures 5A,C) as well as temporal (Figures 5B,D) mutual information of within-ROI voxels increased during stimulation compared with rest, indicating that both the spatial arrangement (across time) and the temporal dynamics (across voxels) of BOLD signals are more similar during stimulation than rest. Increase of spatial mutual information during task is remarkably similar along the repeated stimulation cycles, confirming that the stimulation elicited highly reproducible activation patterns.

## DISCUSSION

In the present work we examined whether and how visual stimulation affects information entropy associated with the BOLD signals both spatially and temporally in the human early visual areas. Since fMRI measures regional averages of the vascular response elicited by an activated cellular population, stimulus-induced changes in BOLD signals may or may not reflect the way sensory information is encoded by neural activity [30]. Functional neuroimaging measurements, such as the BOLD signal, are thought to be correlated differently with specific aspects of neuronal activity [2] as well as of astroglial activity [31, 32]. The limited spatiotemporal resolution of fMRI entails that a certain amount and/or type (e.g., stimulus-dependent) of information is lost. It is likely that information changes depend also on cognitive processes such as visuospatial attention [33] as well as on the relation between the different spatiotemporal scale of stimulus and signal averaging [34]. In an attempt to limit the effect of such relation, in the present work we chose a very simple visual stimulus known to elicit a robust cortical response in area V1. Indeed, the presence of virtually all orientations and spatial frequencies in the visual stimulus are expected to circumvent neuronal stimulus selectivity.

The stimulation-induced decrease in spatial information entropy and the increase in mutual information that we found in the present study is an expected consequence of the stimulus design and of the ROIs selection criteria. It is also consistent with the increased intracluster homogeneity reported elsewhere [18, 19]. However, we also found an increase in temporal information entropy associated with the stimulus, at odds with one previous report [17] but in agreement with another [16]. According to the modern view of entropy variations [35, 36], the change in temporal entropy during stimulation, within a brain area otherwise responding in a uniform way, can be interpreted as the transition from energy being more temporally localized to becoming more temporally dispersed. The latter explanation is valid under the assumption that BOLD signals reflects (though indirectly and nonlinearly) energy consumption [37, 38]. Overall, our results support the notion that within an area where BOLD-related spatial information is reduced due to a uniform stimulation pattern, additional information may be still coded in time domain. The finding that mutual information increases during stimulation both spatially and temporally indicates that stimulated voxels in the relatively small ROI in area V1 (i.e., that is maximally activated by the stimulus) converge to a similar spatial arrangement and temporal dynamics. Therefore, (i) stimulation-induced increase of temporal entropy suggests that signal fluctuations of each voxel in time domain contribute to the information associated with the BOLD response, and (ii) increased mutual information indicates that the time-encoded information gain is obtained via the same temporal dynamics for all voxels.

Similar temporal dynamics for activated voxels is supported by the fact that the temporal entropy increase was almost independent on the extension of the activated ROI. This result is valid within the intrinsic boundaries set by the experimental procedures. They are defined on the small scale by our relatively low spatial resolution (in-plane voxels size 3 mm), and on the large scale by the ROIs selection criteria, that included only activated voxels. Of course, we cannot exclude that distribution profile might actually exhibit different changes at a smaller spatial resolution, for example at the column level, which could be achieved by high-field fMRI [39, 40]. Indeed, the scale-invariant behavior might be different if sensitivity to features inaccessible to our setup (e.g., at the column level) is gained.

In conclusion, in this work we examined the stimulation-dependent changes in spatiotemporal BOLD distribution profile within a uniformly activated functional cluster in human cortical area V1. We found that the voxels responding to stimulation converge to a similar response within the activated area, yet the temporal dynamics of this response is more complex (i.e., encodes more information) during stimulation than rest. In other words, while BOLD maps may be incapable of capturing functional specialization in the space domain due to experimental limitations or by design, information encoding may still be revealed in the temporal domain. Our results indicate that the stimulated state is characterized by a rearrangement of intracluster BOLD responses with corresponding rise in time-encoded information that has not a spatial correlate at the scale of conventional fMRI.

## Supplementary Material

SuppFig **Figure S1. Stimulation appearance and paradigm. (A)** Stimulation consisted of a white-black checkboard presented either in the right or left hemifield. The checkboard was slowly rotating (2 cycles/s) and the direction of rotation was changed with period ranging uniformly between 1 and 3 s. Rest epochs consisted of a full uniform gray field. Subjects were required to fix the central white cross. **(B)** The order of stimulation alternated right-left-rest and left-right-rest within each session, but was fix between subjects, because a fix order was needed for the temporal processing used.
**Figure S2. Space-domain analysis of BOLD distribution in activated primary visual cortex.** Group results for the 5 mm left/right ROIs.
**Figure S3. Space-domain analysis of BOLD distribution in activated primary visual cortex.** Group results for the 6 mm left/right ROIs.
**Figure S4. Space-domain analysis of BOLD distribution in activated primary visual cortex.** Group results for the 8 mm left/right ROIs.
**Figure S5. Space-domain analysis of BOLD distribution in activated primary visual cortex.** Group results for the 9 mm left/right ROIs.
**Figure S6. Space-domain analysis of BOLD distribution in activated primary visual cortex.** Group results for the 10 mm left/right ROIs.
**Figure S7. Time-domain analysis of BOLD distribution in activated primary visual cortex.** Group results for the 5 mm left/right ROIs.
**Figure S8. Time-domain analysis of BOLD distribution in activated primary visual cortex.** Group results for the 6 mm left/right ROIs.
**Figure S9. Time-domain analysis of BOLD distribution in activated primary visual cortex.** Group results for the 8 mm left/right ROIs.
**Figure S10. Time-domain analysis of BOLD distribution in activated primary visual cortex.** Group results for the 9 mm left/right ROIs.
**Figure S11. Time-domain analysis of BOLD distribution in activated primary visual cortex.** Group results for the 10 mm left/right ROIs.
**Figure S12. Correlation between spatial and temporal information entropy in activated primary visual cortex.** Subject results for the 5 mm left/right ROIs.
**Figure S13. Correlation between spatial and temporal information entropy in activated primary visual cortex.** Subject results for the 6 mm left/right ROIs.
**Figure S14. Correlation between spatial and temporal information entropy in activated primary visual cortex.** Subject results for the 8 mm left/right ROIs.
**Figure S15. Correlation between spatial and temporal information entropy in activated primary visual cortex.** Subject results for the 9 mm left/right ROIs.
**Figure S16. Correlation between spatial and temporal information entropy in activated primary visual cortex.** Subject results for the 10 mm left/right ROIs.
**Figure S17. Space- and time-domain mutual information analysis in activated primary visual cortex.** Group results for the 5 mm left/right ROIs.
**Figure S18. Space- and time-domain mutual information analysis in activated primary visual cortex.** Group results for the 6 mm left/right ROIs.
**Figure S19. Space- and time-domain mutual information analysis in activated primary visual cortex.** Group results for the 8 mm left/right ROIs.
**Figure S20. Space- and time-domain mutual information analysis in activated primary visual cortex.** Group results for the 9 mm left/right ROIs.
**Figure S21. Space- and time-domain mutual information analysis in activated primary visual cortex.** Group results for the 10 mm left/right ROIs.

## Figures and Tables

**TABLE 1:** Main characteristics of the activated ROIs as determined by GLM analysis.

Nominal radius (mm)	Voxels count	Mean t-value		p-value	Displacement (mm)		p-value
		Left	Right		Left	Right	
5	19	6.2 ± 1.5	6.7 ± 1.9	0.44	1.9 ± 0.7	1.7 ± 1.1	0.56
6	29	5.6 ± 1.1	6.0 ± 1.5	0.43	2.2 ± 0.7	2.4 ± 1.5	0.64
7	39	5.2 ± 1.1	5.5 ± 1.1	0.48	2.7 ± 1.1	2.9 ± 1.5	0.68
8	65	4.8 ± 0.7	5.0 ± 0.7	0.48	4.0 ± 1.9	3.8 ± 2.2	0.79
9	87	4.5 ± 0.7	4.7 ± 0.7	0.49	5.1 ± 2.6	4.5 ± 2.6	0.53
10	121	4.3 ± 0.7	4.5 ± 0.7	0.49	6.9 ± 3.4	6.0 ± 3.0	0.45

Displacement is determined as the distance between coordinate of peak t-value and center of mass within the ROI.

ROIs are built according to the procedure detailed in the ROI definition section within methods. The average value for the peak t-value across all subjects (mean ± sd, n = 15) is 13.9 ± 3.0 for the left ROI and 15.5 ± 4.1 for the right ROI. Values, expressed as mean ± sd (n = 15), are not statistically different between left and right ROIs.

**TABLE 2:** Stimulation-induced changes in BOLD distribution parameters as a function of ROI size.

Parameter	Nominal radius (mm)						F
	5	6	7	8	9	10	
**LEFT ROI**							
**Spatial**							
Mean (×10^−3^)	36.7 ± 9.3*	32.0 ± 6.9*	29.4 ± 5.4*	25.8 ± 3.9*	24.3 ± 3.1*	23.0 ± 2.3*	20.65*
Variance (×10^−4^)	2.9 ± 2.3*	2.5 ± 1.2*	2.1 ± 1.2*	1.6 ± 0.8*	1.5 ± 0.4*	1.3 ± 0.4*	8.57*
Skewness (×10^−2^)	9.0 ± 14.7*	9.9 ± 12.0*	11.1 ± 10.1*	12.4 ± 6.9*	14.0 ± 6.2*	13.2 ± 5.4*	0.95
Kurtosis (×10^−2^)	16.1 ± 37.2*	16.2 ± 31.7	20.0 ± 31.4*	27.5 ± 23.6*	29.1 ± 22.5*	27.8 ± 20.9*	0.90
Normality (×10^−2^)	−3.6 ± 10.1	−4.6 ± 7.3*	−6.5 ± 6.9*	−7.0 ± 5.4*	−8.3 ± 5.0*	−7.4 ± 3.5*	1.72
Entropy (×10^−3^)	−10.9 ± 26.3	−8.0 ± 17.4	−9.1 ± 14.3*	−9.7 ± 9.3*	−9.8 ± 8.1*	−8.6 ± 6.6*	0.06
**Temporal**							
Mean (×10^−3^)	37.3 ± 8.9*	32.5 ± 5.0*	29.7 ± 3.9*	25.9 ± 2.7*	24.4 ± 1.9*	23.2 ± 1.5*	18.41*
Variance (×10^−4^)	0.4 ± 1.9	0.2 ± 1.9	0.2 ± 1.5	0.1 ± 1.2	0.1 ± 0.8	0.0 ± 0.8	0.91
Skewness (×10^−2^)	−3.0 ± 5.8	−1.3 ± 5.8	−0.1 ± 4.3	1.2 ± 3.1	1.4 ± 2.7	1.8 ± 2.7	2.55
Kurtosis (×10^−2^)	−36.7 ± 16.6*	−31.8 ± 15.9*	−24.9 ± 13.6*	−20.1 ± 9.7*	−20.0 ± 9.3*	−19.4 ± 8.1*	3.65
Normality (×10^−2^)	5.7 ± 7.7	6.0 ± 7.7	4.4 ± 6.2	4.1 ± 5.0	3.7 ± 4.6	3.7 ± 3.9*	0.94
Entropy (×10^−3^)	29.5 ± 11.2*	26.9 ± 10.1*	23.7 ± 9.3*	21.2 ± 6.2*	21.0 ± 5.8*	19.9 ± 5.4*	2.49

**RIGHT ROI**							
**Spatial**							
Mean (×10^−3^)	37.9 ± 10.1*	33.1 ± 6.9*	29.9 ± 5.4*	26.3 ± 3.9*	24.7 ± 3.1*	23.7 ± 2.3*	17.64*
Variance (×10^−4^)	2.6 ± 2.3*	2.0 ± 1.5*	1.6 ± 1.2*	1.2 ± 0.8*	1.0 ± 0.4*	0.7 ± 0.4*	14.28*
Skewness (×10^−2^)	8.9 ± 13.9*	8.4 ± 10.1*	11.5 ± 9.3*	10.2 ± 6.9*	8.3 ± 5.8*	8.4 ± 5.4*	0.53
Kurtosis (×10^−2^)	15.4 ± 37.9	16.2 ± 31.7	24.3 ± 29.0*	24.3 ± 23.2*	20.9 ± 20.9*	20.4 ± 20.1	0.38
Normality (×10^−2^)	−7.0 ± 10.1*	−4.9 ± 6.9*	−7.4 ± 6.6*	−6.0 ± 5.0*	−4.1 ± 4.6	−3.3 ± 3.5	0.94
Entropy (×10^−3^)	−16.0 ± 26.3*	−12.3 ± 17.0*	−13.3 ± 14.3*	−9.7 ± 10.1*	−8.5 ± 8.1*	−5.1 ± 6.6	1.68
**Temporal**							
Mean (×10^−3^)	38.4 ± 6.9*	33.4 ± 3.9*	30.2 ± 3.1*	26.5 ± 2.3*	24.8 ± 1.9*	23.8 ± 1.5*	25.05*
Variance (×10^−4^)	0.8 ± 1.9	0.7 ± 1.5	0.6 ± 1.2	0.6 ± 0.8	0.7 ± 0.8*	0.8 ± 1.2	0.91
Skewness (×10^−2^)	4.2 ± 6.9	3.9 ± 5.4	3.8 ± 3.9*	5.1 ± 3.5*	5.2 ± 3.1*	5.0 ± 2.7*	0.33
Kurtosis (×10^−2^)	−4.5 ± 21.7	−5.5 ± 16.3	−6.8 ± 13.2	−5.1 ± 10.8	−4.9 ± 9.7	−4.5 ± 7.7	0.08
Normality (×10^−2^)	4.7 ± 9.3	3.3 ± 8.1	4.1 ± 5.0	2.9 ± 4.6	2.3 ± 4.6	3.0 ± 3.5	0.26
Entropy (×10^−3^)	13.6 ± 12.4*	15.6 ± 9.3*	15.2 ± 9.7*	14.0 ± 7.4*	13.6 ± 6.2*	13.4 ± 5.0*	0.18

Changes are calculated as differences (stimulation-rest) of intersubject averages. All values (except for F-values) are expressed as mean ± sd (n = 15) in the relevant units used in Figures 2, 3
