# Supplementary material for: Temporal Information Entropy of the Blood-Oxygenation Level-Dependent Signals Increases in the Activated Human Primary Visual Cortex
Source: Front Phys. Author manuscript; Available in PMC 2017 Apr 25. (PMC5404702; doi:10.3389/fphy.2017.00007)
Supplement: SuppFig — Figure S1. Stimulation appearance and paradigm. (A) Stimulation consisted of a white-black checkboard presented either in the right or left hemifield. The checkboard was slowly rotating (2 cycles/s) and the direction of rotation was changed with period ranging uniformly between 1 and 3 s. Rest epochs consisted of a full uniform gray field. Subjects were required to fix the central white cross. (B) The order of stimulation alternated right-left-rest and left-right-rest within each session, but was fix between subjects, because a fix order was needed for the temporal processing used. Figure S2. Space-domain analysis of BOLD distribution in activated primary visual cortex. Group results for the 5 mm left/right ROIs. Figure S3. Space-domain analysis of BOLD distribution in activated primary visual cortex. Group results for the 6 mm left/right ROIs. Figure S4. Space-domain analysis of BOLD distribution in activated primary visual cortex. Group results for the 8 mm left/right ROIs. Figure S5. Space-domain analysis of BOLD distribution in activated primary visual cortex. Group results for the 9 mm left/right ROIs. Figure S6. Space-domain analysis of BOLD distribution in activated primary visual cortex. Group results for the 10 mm left/right ROIs. Figure S7. Time-domain analysis of BOLD distribution in activated primary visual cortex. Group results for the 5 mm left/right ROIs. Figure S8. Time-domain analysis of BOLD distribution in activated primary visual cortex. Group results for the 6 mm left/right ROIs. Figure S9. Time-domain analysis of BOLD distribution in activated primary visual cortex. Group results for the 8 mm left/right ROIs. Figure S10. Time-domain analysis of BOLD distribution in activated primary visual cortex. Group results for the 9 mm left/right ROIs. Figure S11. Time-domain analysis of BOLD distribution in activated primary visual cortex. Group results for the 10 mm left/right ROIs. Figure S12. Correlation between spatial and temporal information entropy [file NIHMS855114-supplement-SuppFig.pdf]

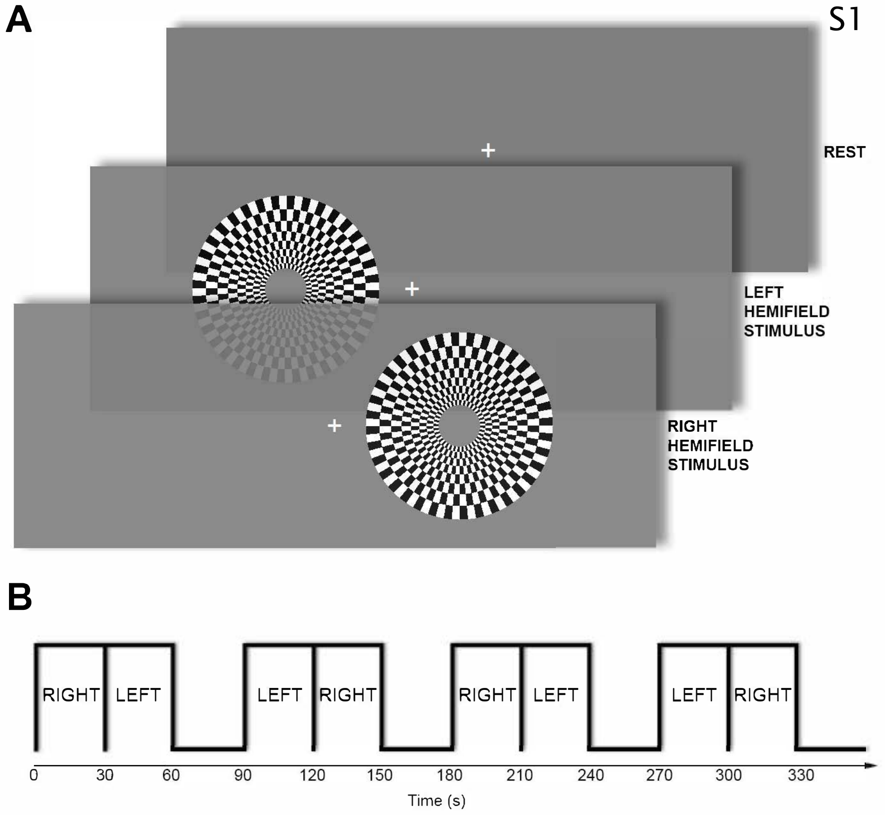
A
S1
REST
LEFT HEMIFIELD STIMULUS
RIGHT HEMIFIELD STIMULUS
B
RIGHT
LEFT
LEFT
RIGHT
RIGHT
LEFT
LEFT
RIGHT
0
30
60
90
120
150
180
210
240
270
300
330
Time (s)

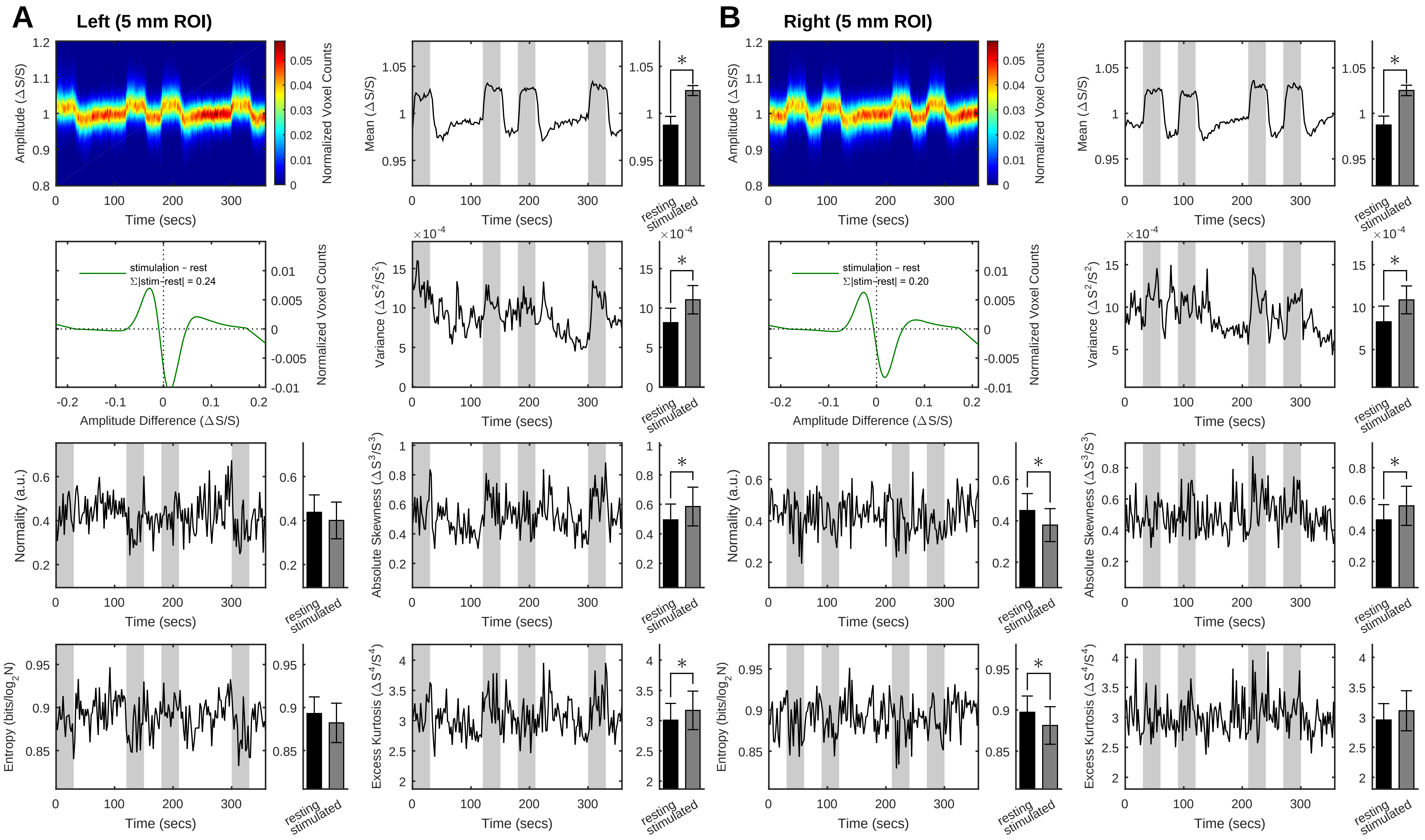
A
Left (5 mm ROI)
B
Right (5 mm ROI)
Amplitude (ΔS/S)
Normalized Voxel Counts
Time (secs)
Mean (ΔS/S)
resting
stimulated
*
stimulation – rest
Σ|stim–rest| = 0.24
Σ|stim–rest| = 0.20
Amplitude Difference (ΔS/S)
Variance (ΔS²/S²)
Normality (a.u.)
Absolute Skewness (ΔS³/S³)
Entropy (bits/log₂N)
Excess Kurtosis (ΔS⁴/S⁴)

S2

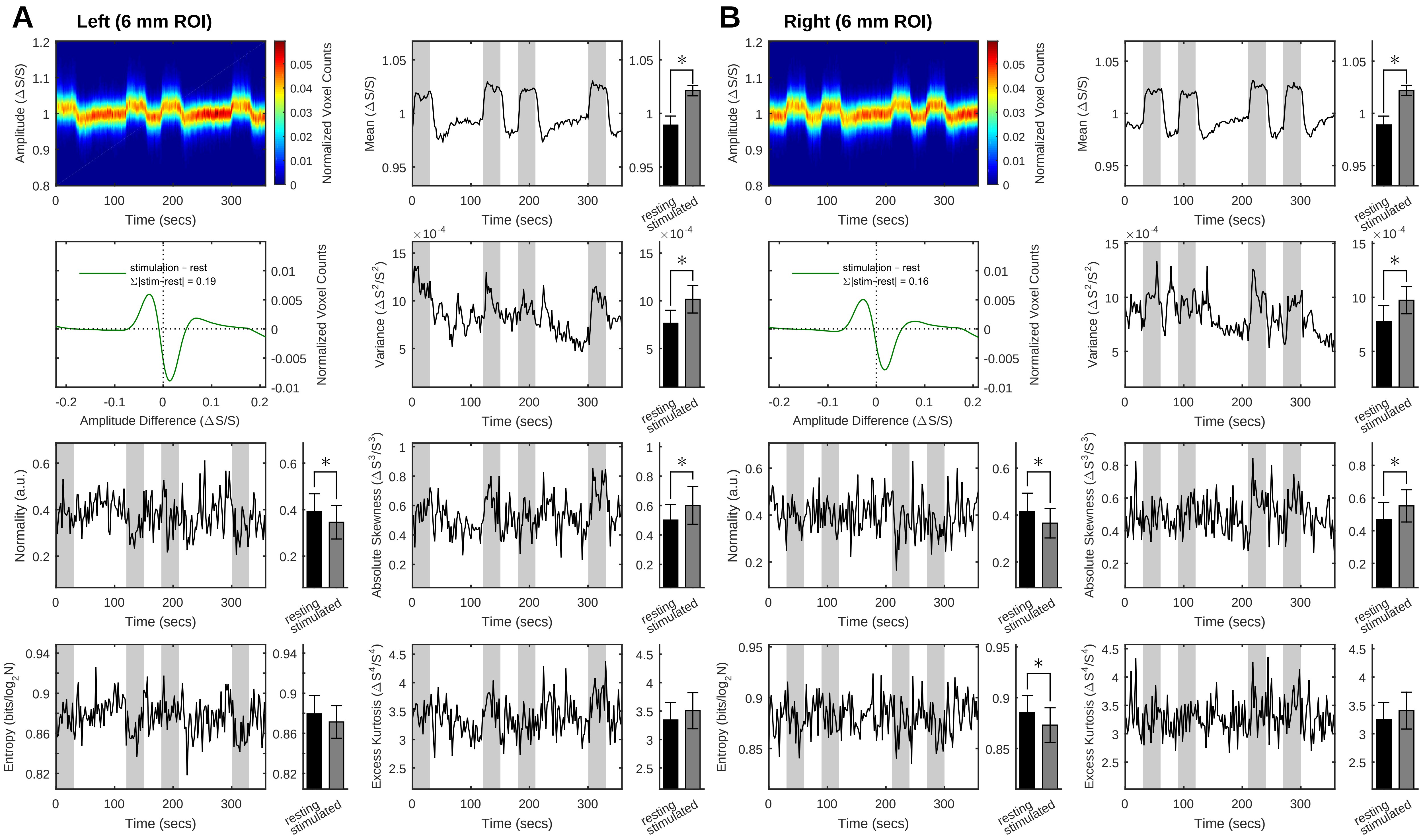

A
Left (6 mm ROI)
B
Right (6 mm ROI)
Amplitude (ΔS/S)
Normalized Voxel Counts
Time (secs)
Mean (ΔS/S)
resting
stimulated
*
stimulation − rest
Σ|stim−rest| = 0.19
Σ|stim−rest| = 0.16
Amplitude Difference (ΔS/S)
Variance (ΔS²/S²)
×10⁻⁴
Normality (a.u.)
Absolute Skewness (ΔS³/S³)
Entropy (bits/log₂N)
Excess Kurtosis (ΔS⁴/S⁴)

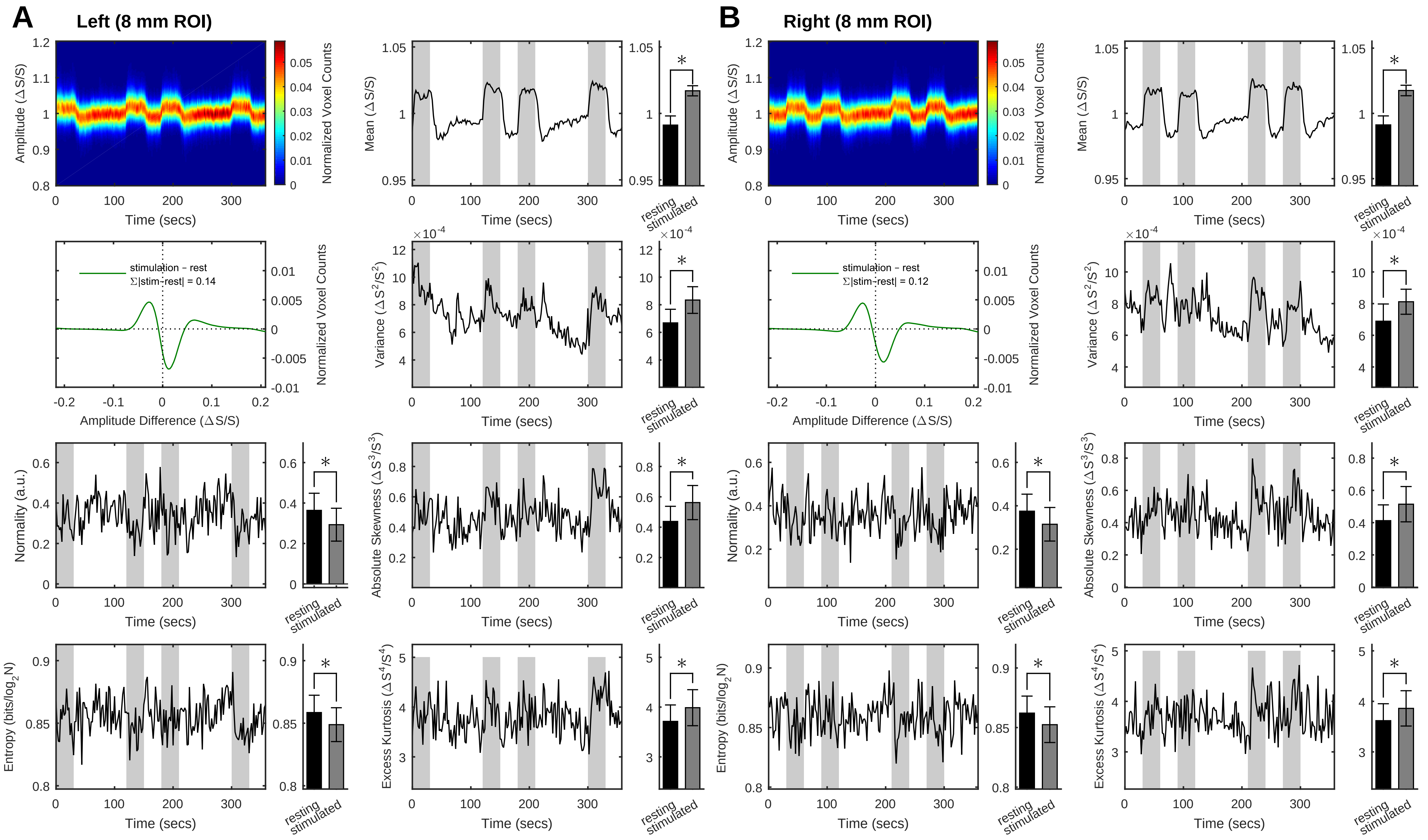
A
Left (8 mm ROI)
B
Right (8 mm ROI)
Amplitude (ΔS/S)
Normalized Voxel Counts
Time (secs)
Mean (ΔS/S)
resting
stimulated
*
stimulation – rest
Σ|stim–rest| = 0.14
Σ|stim–rest| = 0.12
Amplitude Difference (ΔS/S)
Variance (ΔS²/S²)
×10⁻⁴
Normality (a.u.)
Absolute Skewness (ΔS³/S³)
Entropy (bits/log₂N)
Excess Kurtosis (ΔS⁴/S⁴)

S4

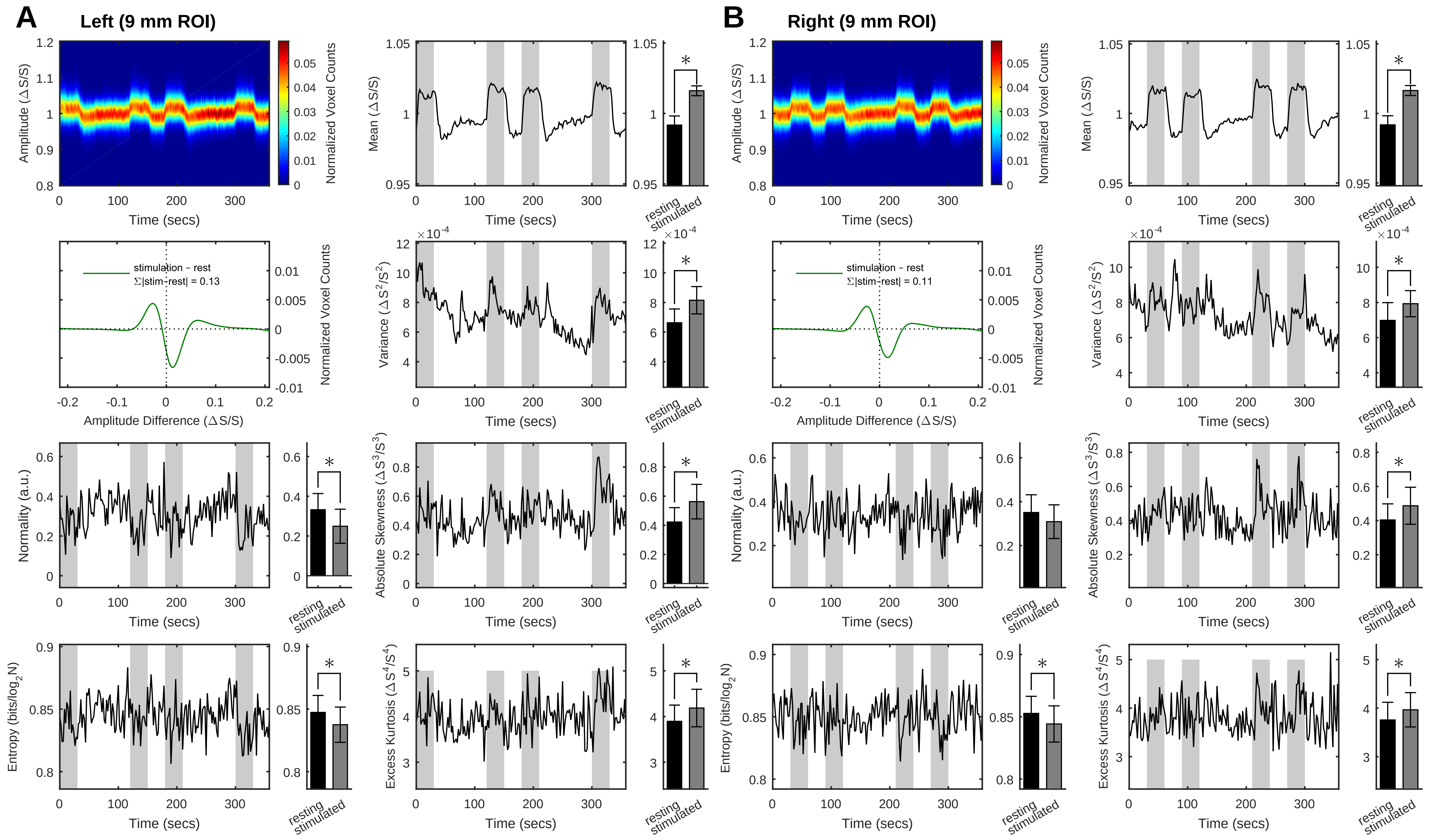

A
Left (9 mm ROI)
B
Right (9 mm ROI)
Amplitude (ΔS/S)
Normalized Voxel Counts
Time (secs)
Mean (ΔS/S)
resting
stimulated
stimulation − rest
Σ|stim−rest| = 0.13
Σ|stim−rest| = 0.11
Amplitude Difference (ΔS/S)
Variance (ΔS²/S²)
Normality (a.u.)
Absolute Skewness (ΔS³/S³)
Entropy (bits/log₂N)
Excess Kurtosis (ΔS⁴/S⁴)

S5

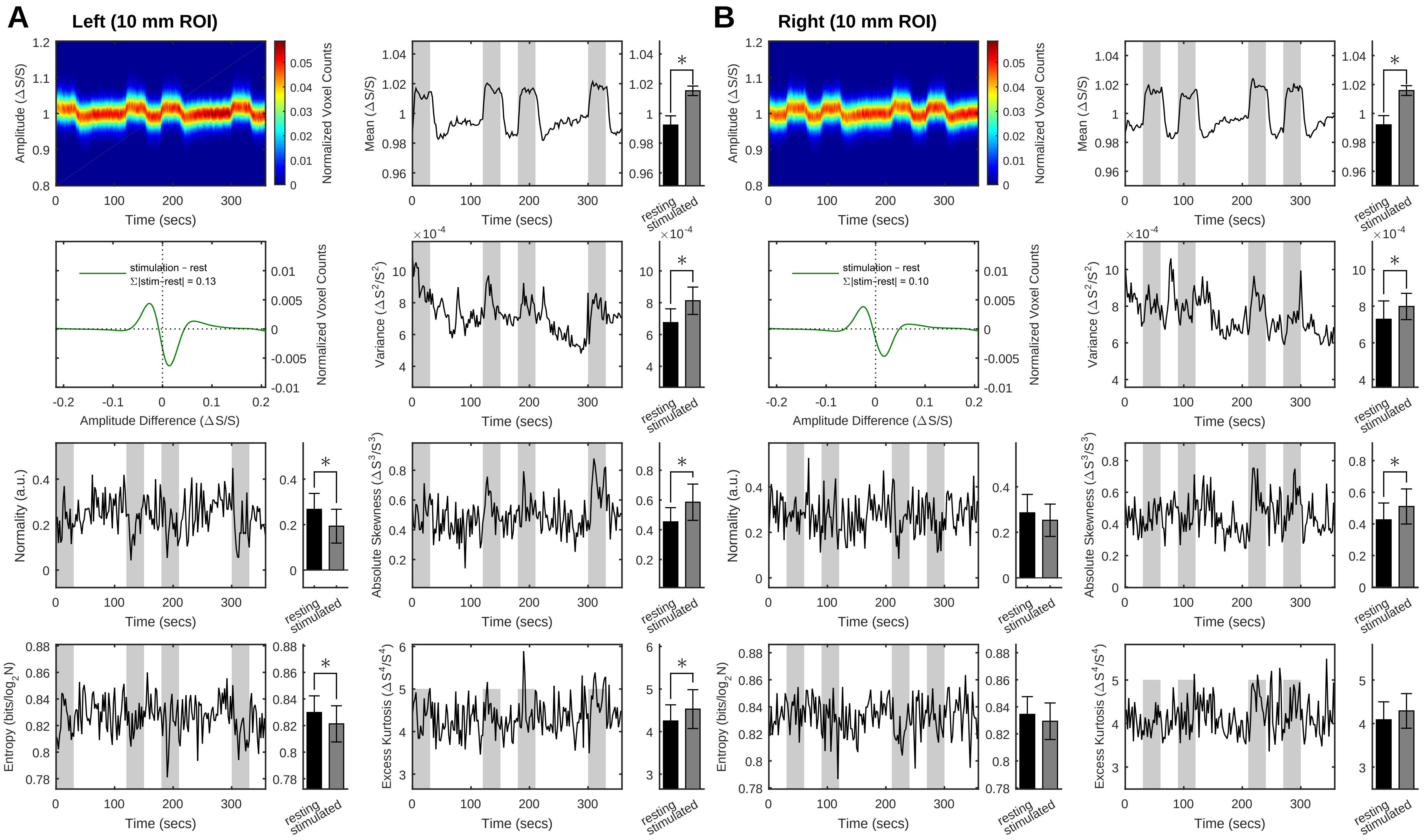
A
Left (10 mm ROI)
B
Right (10 mm ROI)
Amplitude (ΔS/S)
Time (secs)
Normalized Voxel Counts
Mean (ΔS/S)
resting
stimulated
*
stimulation − rest
Σ|stim−rest| = 0.13
Σ|stim−rest| = 0.10
Amplitude Difference (ΔS/S)
Variance (ΔS²/S²)
×10⁻⁴
Normality (a.u.)
Absolute Skewness (ΔS³/S³)
Entropy (bits/log₂N)
Excess Kurtosis (ΔS⁴/S⁴)

S6

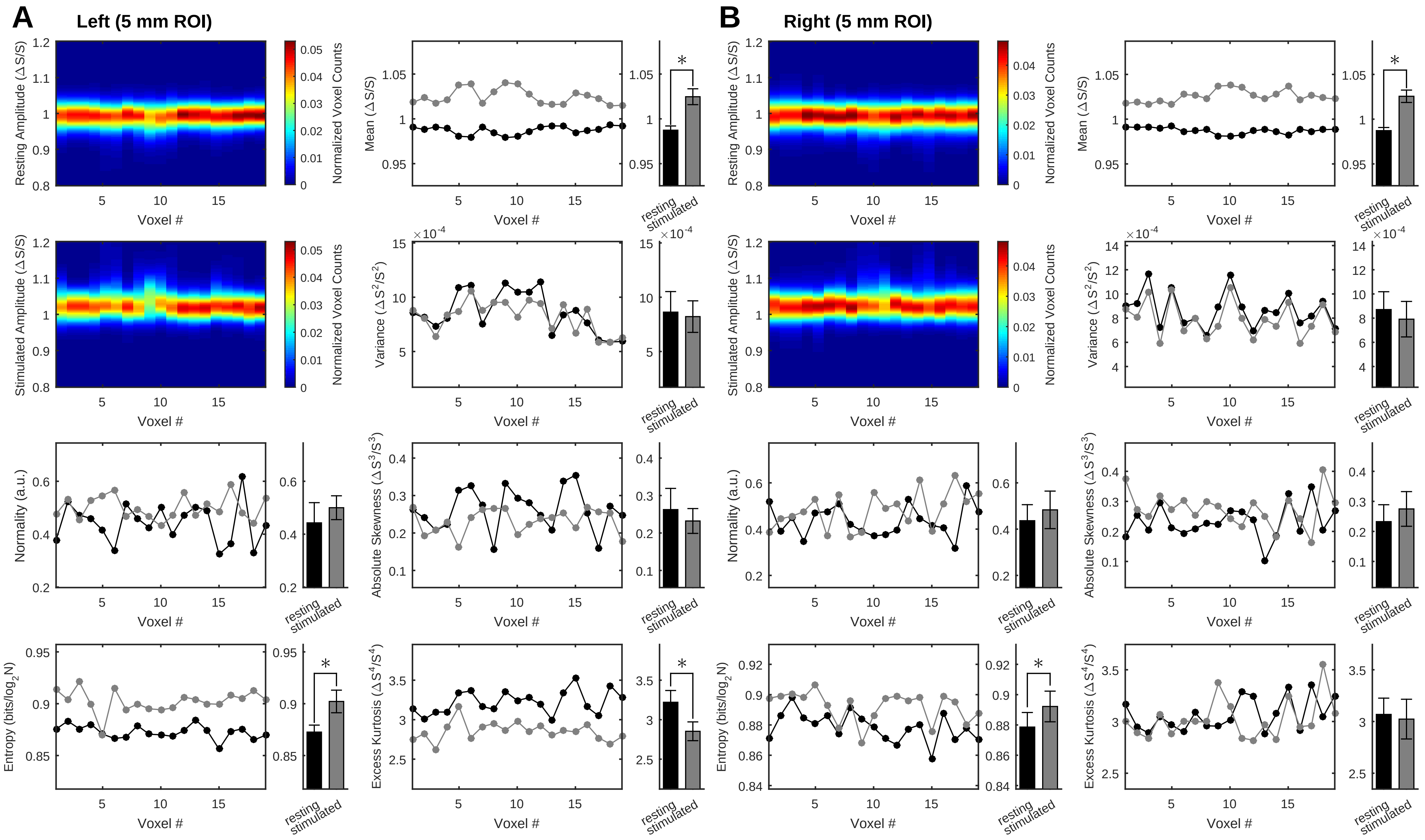
A
Left (5 mm ROI)
B
Right (5 mm ROI)
Resting Amplitude (ΔS/S)
Stimulated Amplitude (ΔS/S)
Normalized Voxel Counts
Voxel #
Mean (ΔS/S)
Variance (ΔS²/S²)
Normality (a.u.)
Absolute Skewness (ΔS³/S³)
Entropy (bits/log₂N)
Excess Kurtosis (ΔS⁴/S⁴)
resting
stimulated
*

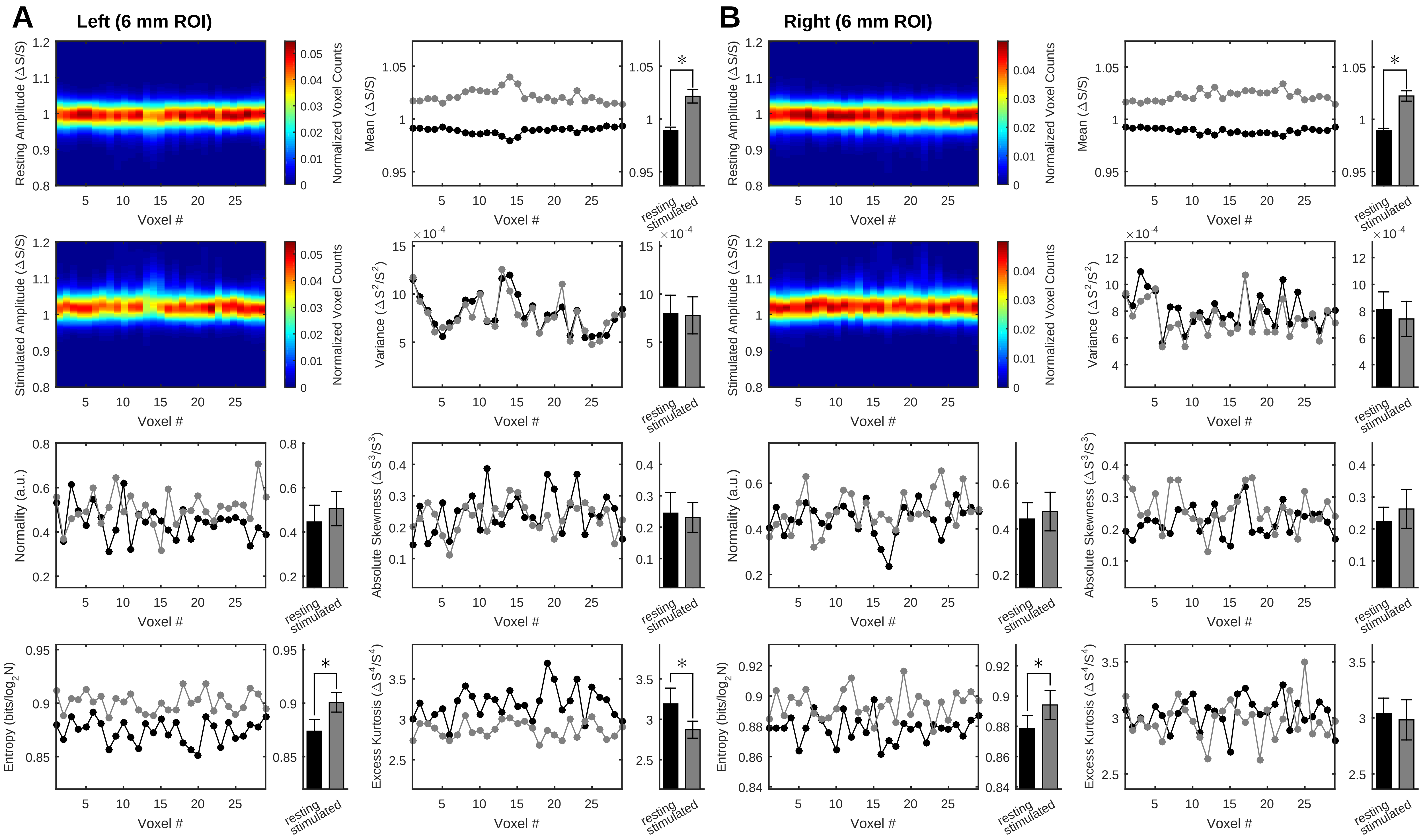
A
Left (6 mm ROI)
B
Right (6 mm ROI)
Resting Amplitude (ΔS/S)
Stimulated Amplitude (ΔS/S)
Normalized Voxel Counts
Mean (ΔS/S)
Variance (ΔS²/S²)
Normality (a.u.)
Absolute Skewness (ΔS³/S³)
Entropy (bits/log₂N)
Excess Kurtosis (ΔS⁴/S⁴)
Voxel #
resting
stimulated
×10⁻⁴
*

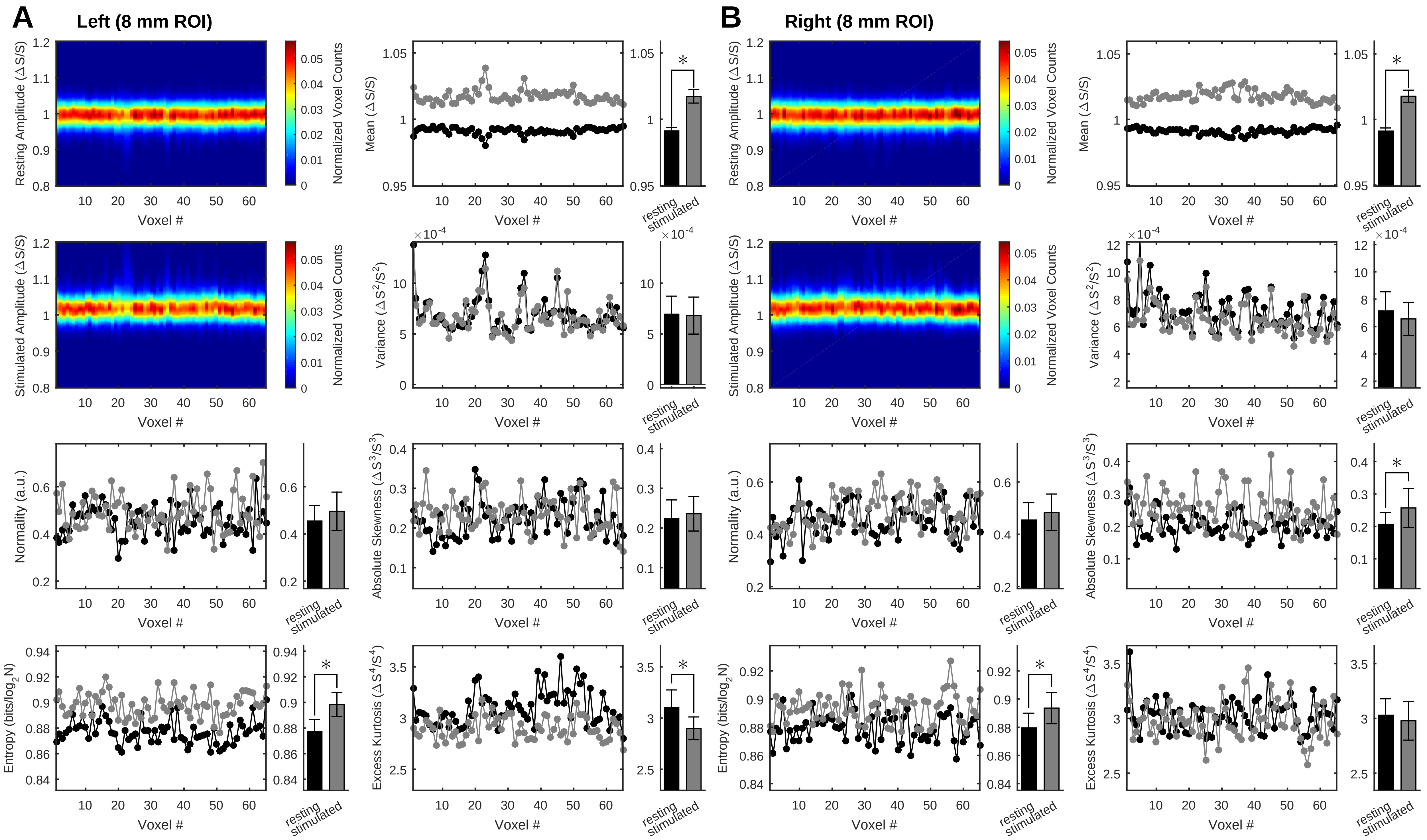
A
Left (8 mm ROI)
B
Right (8 mm ROI)
Resting Amplitude (ΔS/S)
Stimulated Amplitude (ΔS/S)
Normalized Voxel Counts
Mean (ΔS/S)
Variance (ΔS²/S²)
×10⁻⁴
Normality (a.u.)
Absolute Skewness (ΔS³/S³)
Entropy (bits/log₂N)
Excess Kurtosis (ΔS⁴/S⁴)
Voxel #
resting
stimulated
*

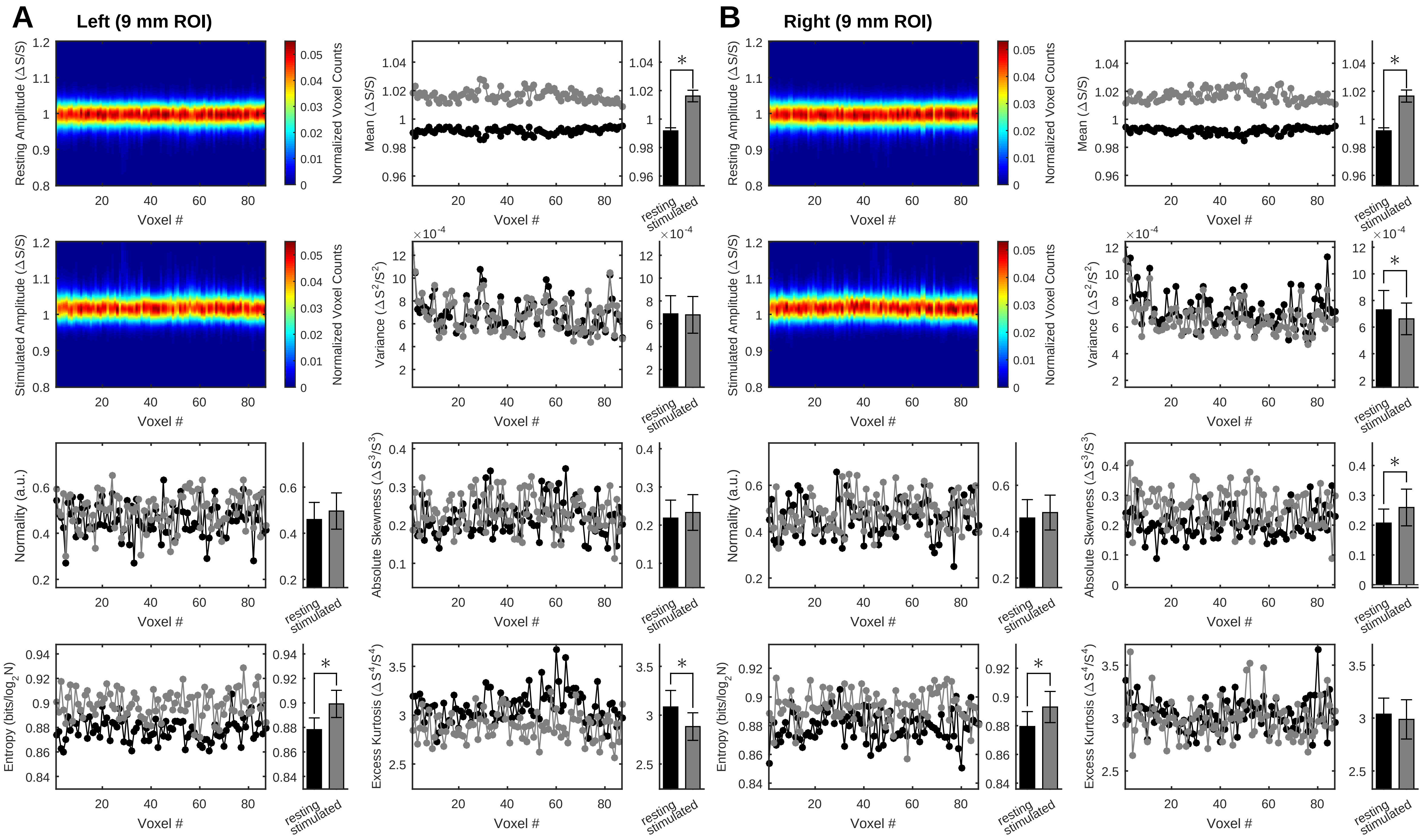
A
Left (9 mm ROI)
B
Right (9 mm ROI)
Resting Amplitude (ΔS/S)
Stimulated Amplitude (ΔS/S)
Normalized Voxel Counts
Voxel #
Mean (ΔS/S)
Variance (ΔS²/S²)
×10⁻⁴
Normality (a.u.)
Absolute Skewness (ΔS³/S³)
Entropy (bits/log₂N)
Excess Kurtosis (ΔS⁴/S⁴)
resting
stimulated
*

S10

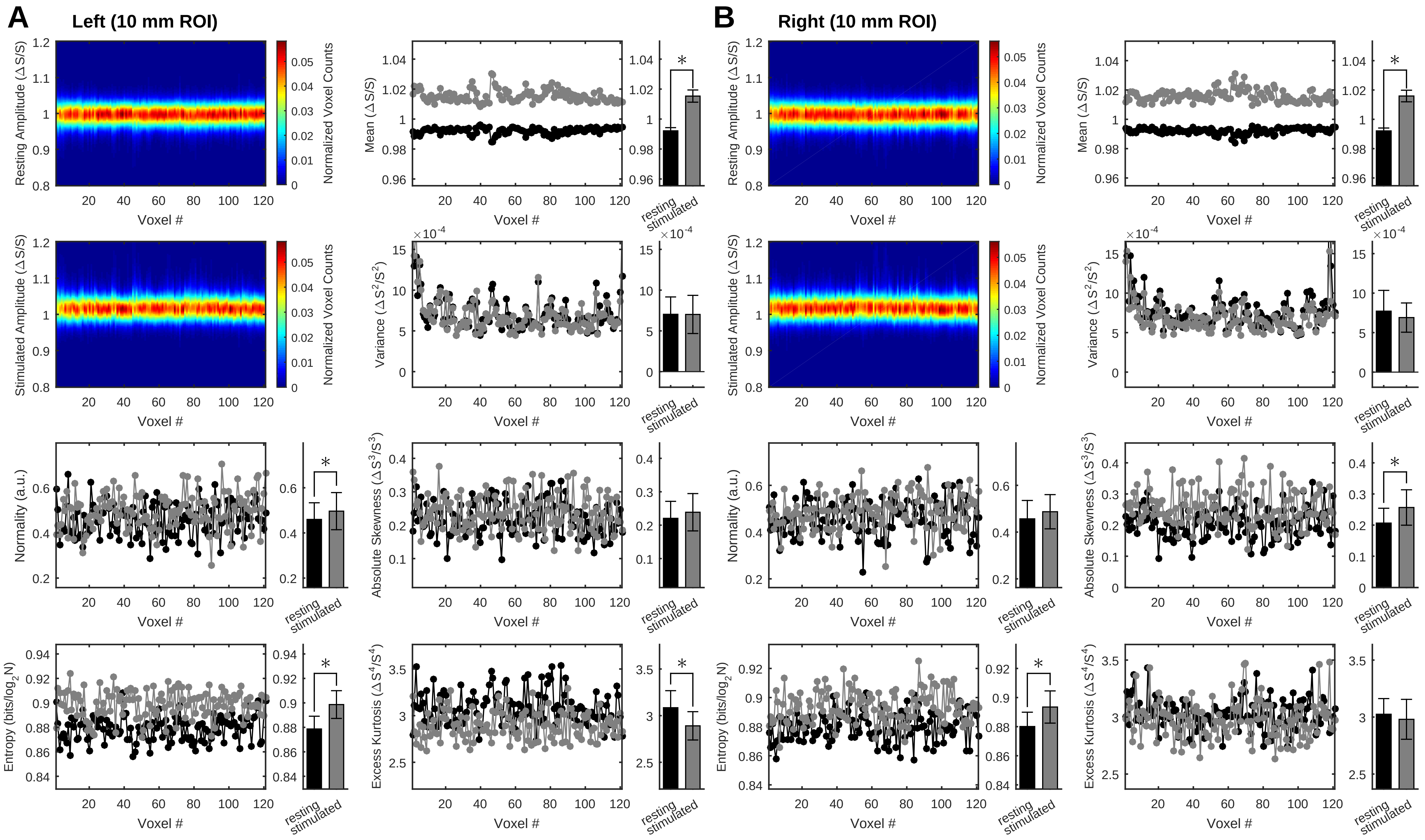
A
Left (10 mm ROI)
B
Right (10 mm ROI)
Resting Amplitude (ΔS/S)
Stimulated Amplitude (ΔS/S)
Normalized Voxel Counts
Voxel #
Mean (ΔS/S)
Variance (ΔS²/S²)
×10⁻⁴
Normality (a.u.)
Absolute Skewness (ΔS³/S³)
Entropy (bits/log₂N)
Excess Kurtosis (ΔS⁴/S⁴)
resting
stimulated
*

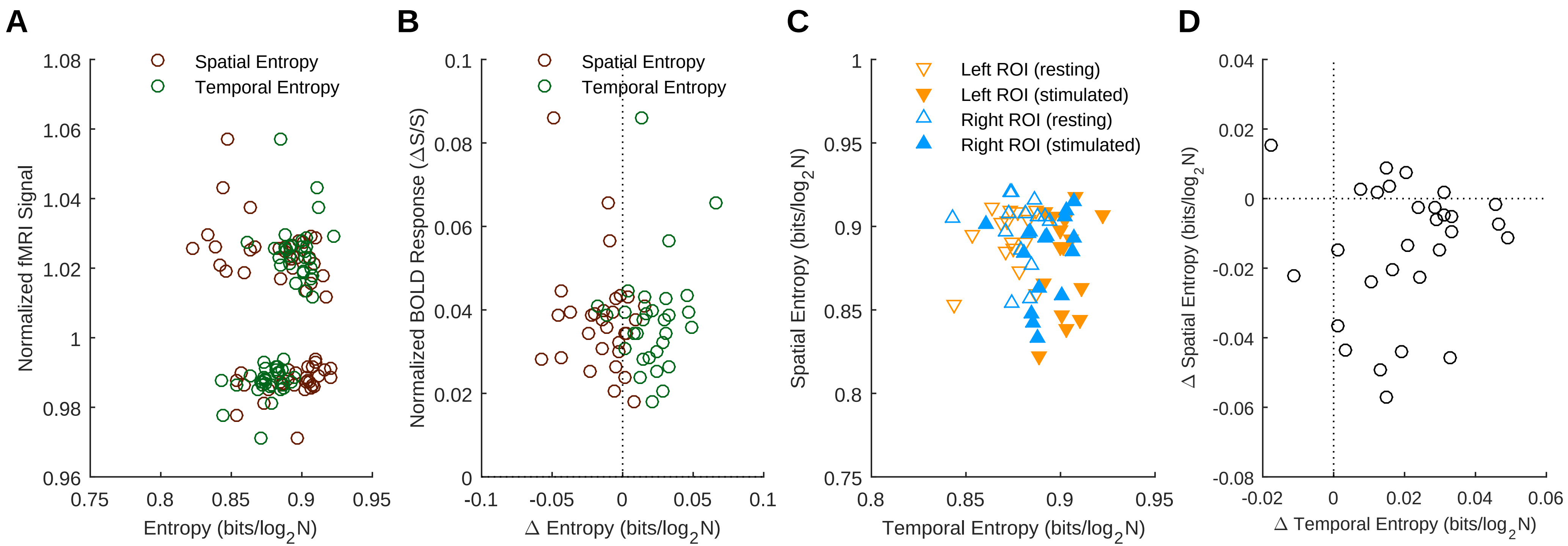
A
Spatial Entropy
Temporal Entropy
Normalized fMRI Signal
Entropy (bits/log2N)
B
Spatial Entropy
Temporal Entropy
Normalized BOLD Response (ΔS/S)
Δ Entropy (bits/log2N)
C
Left ROI (resting)
Left ROI (stimulated)
Right ROI (resting)
Right ROI (stimulated)
Spatial Entropy (bits/log2N)
Temporal Entropy (bits/log2N)
D
Δ Spatial Entropy (bits/log2N)
Δ Temporal Entropy (bits/log2N)

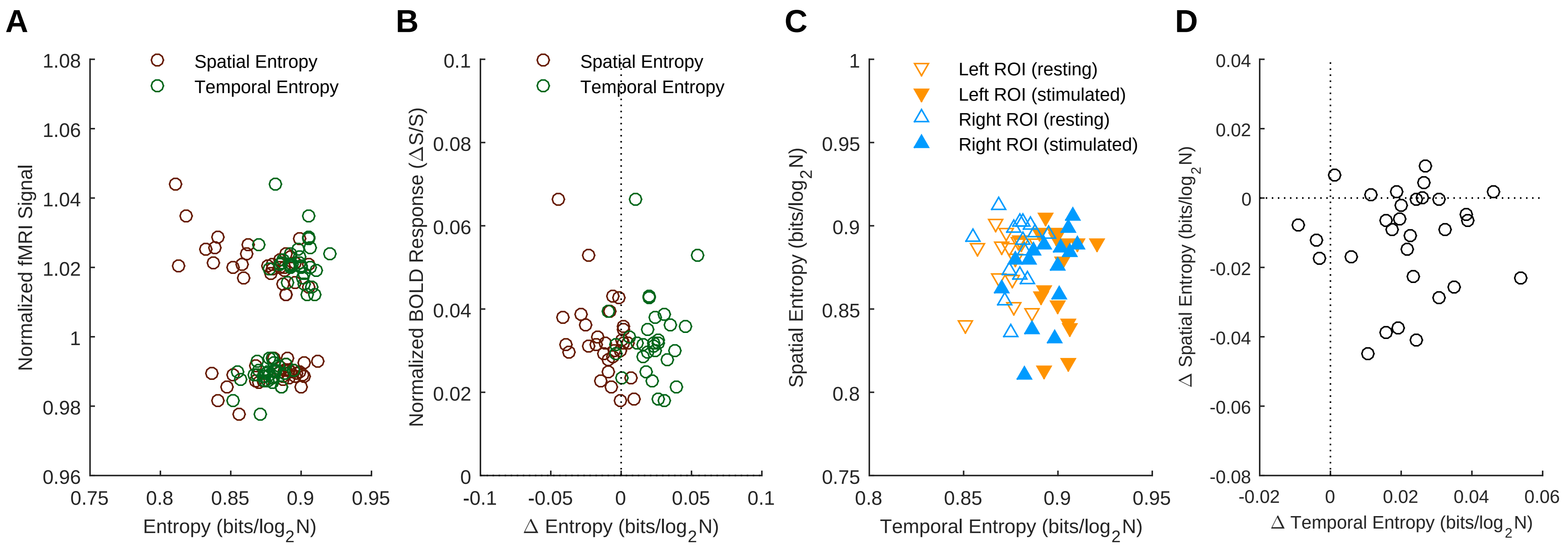
A
B
C
D
Spatial Entropy
Temporal Entropy
Normalized fMRI Signal
Entropy (bits/log$_2$N)
Normalized BOLD Response (ΔS/S)
Δ Entropy (bits/log$_2$N)
Left ROI (resting)
Left ROI (stimulated)
Right ROI (resting)
Right ROI (stimulated)
Spatial Entropy (bits/log$_2$N)
Temporal Entropy (bits/log$_2$N)
Δ Spatial Entropy (bits/log$_2$N)
Δ Temporal Entropy (bits/log$_2$N)

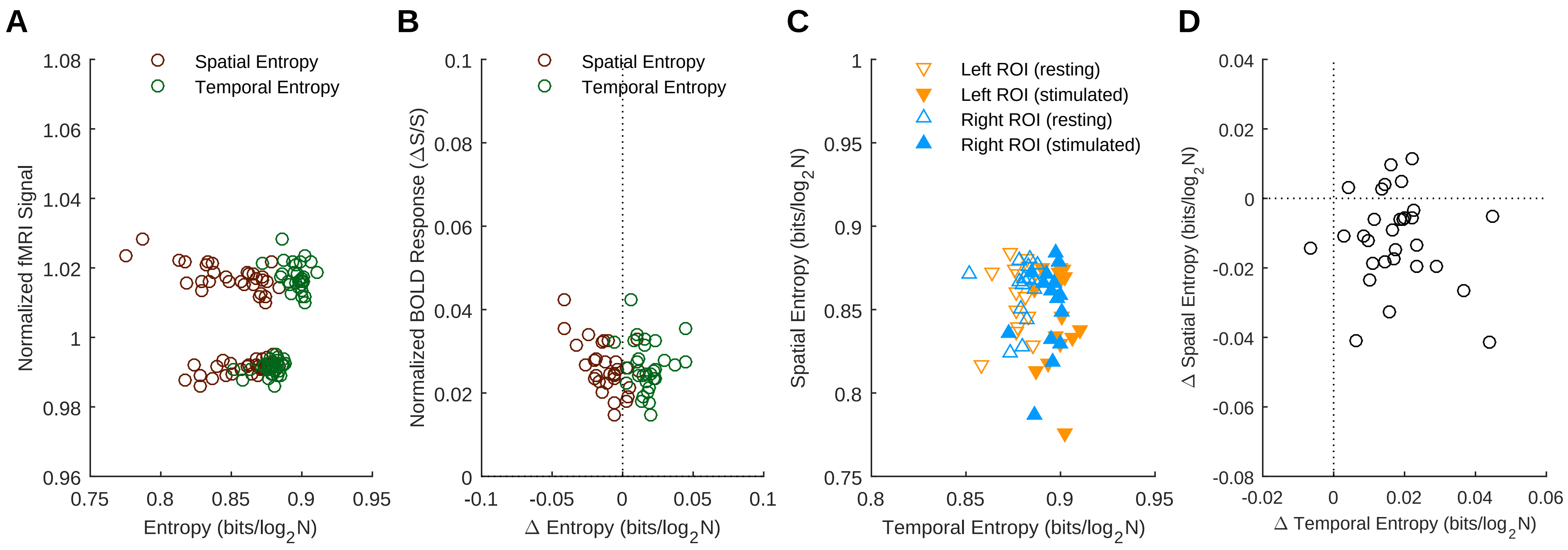
A
B
C
D
Spatial Entropy
Temporal Entropy
Normalized fMRI Signal
Entropy (bits/log2N)
Normalized BOLD Response (ΔS/S)
Δ Entropy (bits/log2N)
Left ROI (resting)
Left ROI (stimulated)
Right ROI (resting)
Right ROI (stimulated)
Spatial Entropy (bits/log2N)
Temporal Entropy (bits/log2N)
Δ Spatial Entropy (bits/log2N)
Δ Temporal Entropy (bits/log2N)

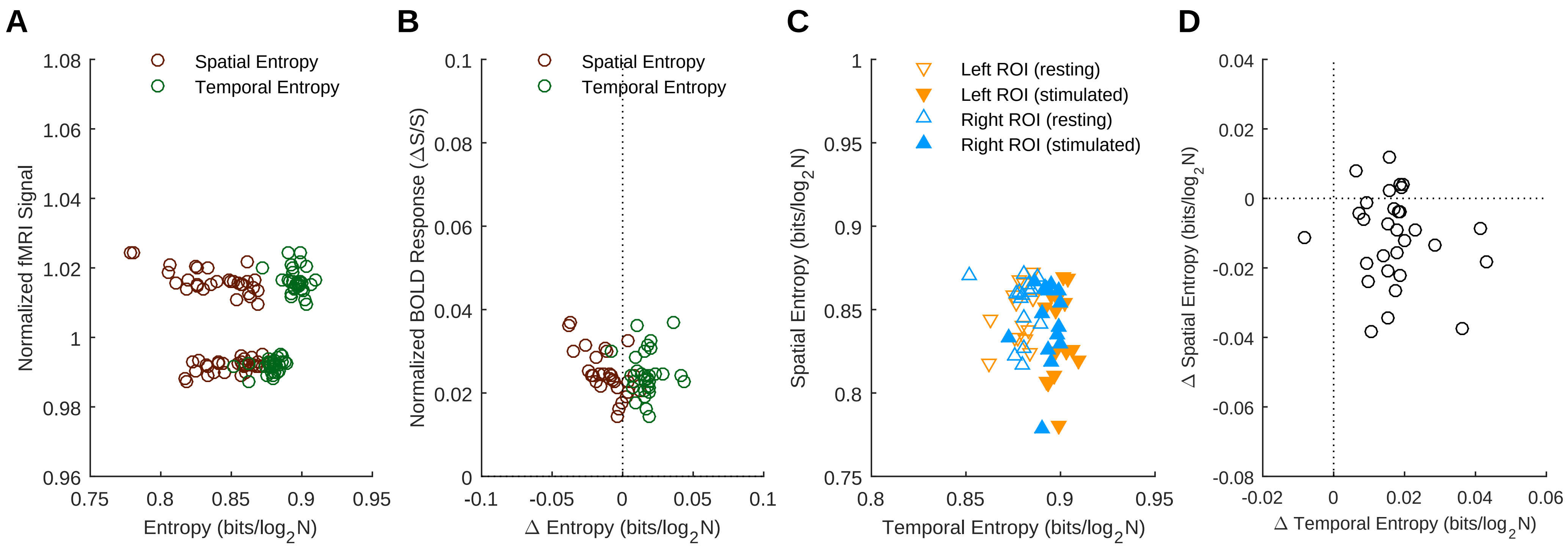

A
Spatial Entropy
Temporal Entropy
Normalized fMRI Signal
Entropy (bits/log$_2$N)
B
Spatial Entropy
Temporal Entropy
Normalized BOLD Response (ΔS/S)
Δ Entropy (bits/log$_2$N)
C
Left ROI (resting)
Left ROI (stimulated)
Right ROI (resting)
Right ROI (stimulated)
Spatial Entropy (bits/log$_2$N)
Temporal Entropy (bits/log$_2$N)
D
Δ Spatial Entropy (bits/log$_2$N)
Δ Temporal Entropy (bits/log$_2$N)

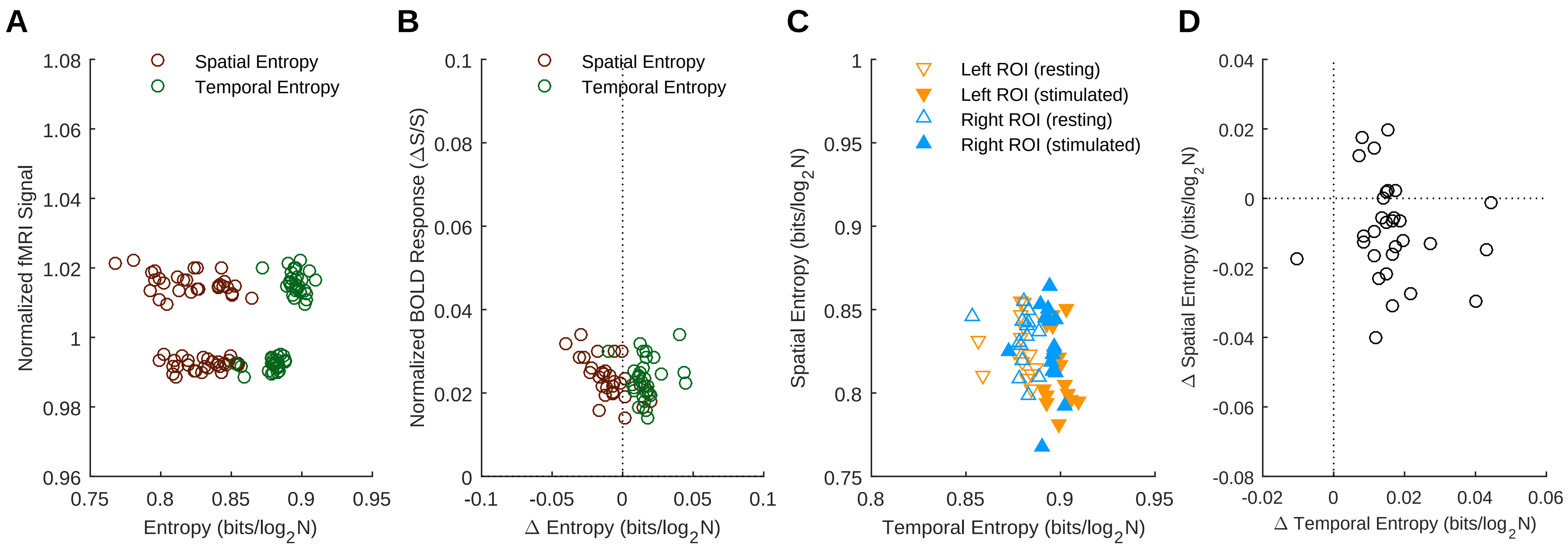
A
B
C
D
Spatial Entropy
Temporal Entropy
Normalized fMRI Signal
Entropy (bits/log2N)
Normalized BOLD Response (ΔS/S)
Δ Entropy (bits/log2N)
Left ROI (resting)
Left ROI (stimulated)
Right ROI (resting)
Right ROI (stimulated)
Spatial Entropy (bits/log2N)
Temporal Entropy (bits/log2N)
Δ Spatial Entropy (bits/log2N)
Δ Temporal Entropy (bits/log2N)

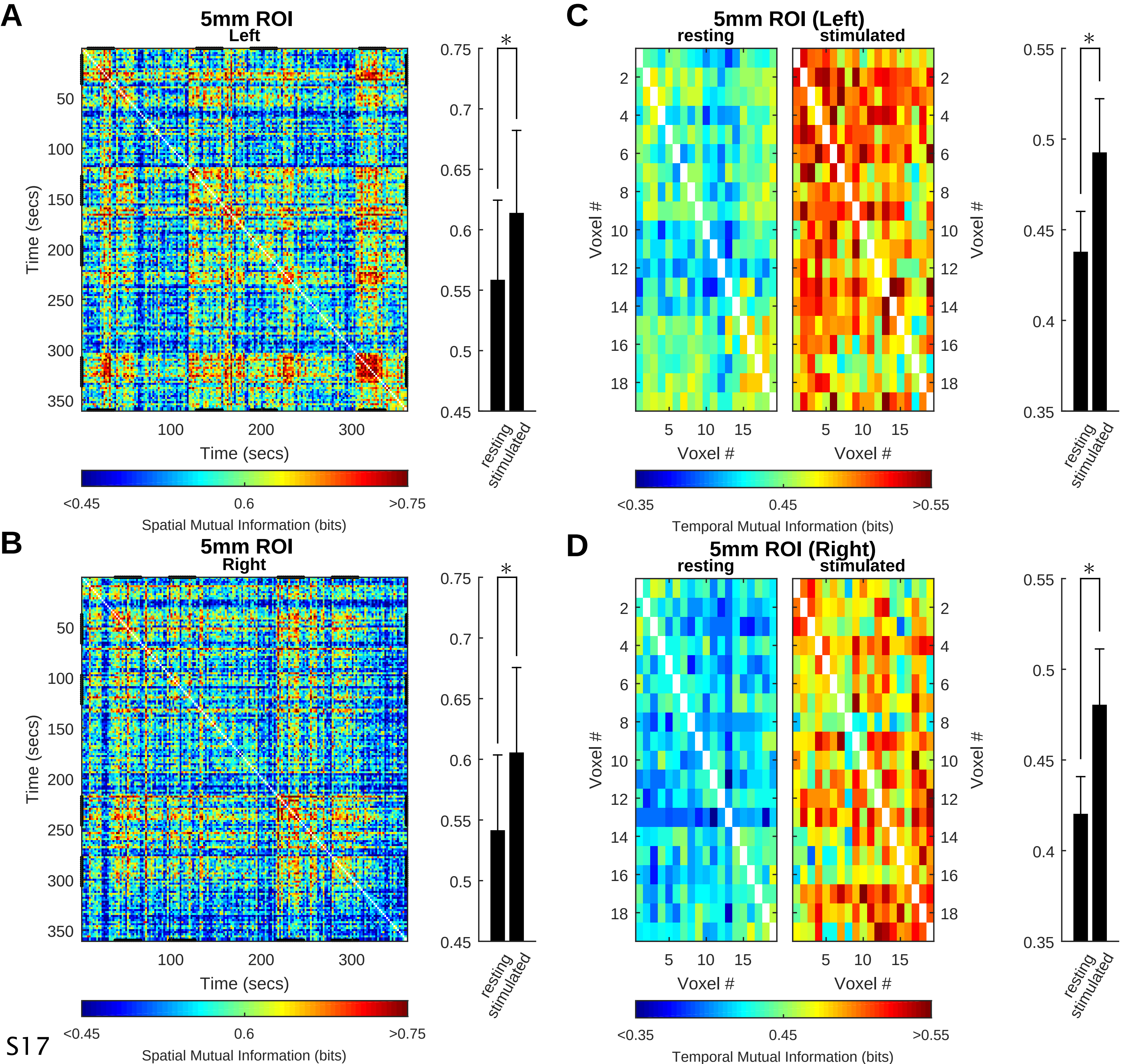
A
5mm ROI
Left
Time (secs)
Time (secs)
resting
stimulated
<0.45
0.6
>0.75
Spatial Mutual Information (bits)
B
5mm ROI
Right
Time (secs)
Time (secs)
resting
stimulated
<0.45
0.6
>0.75
Spatial Mutual Information (bits)
C
5mm ROI (Left)
resting
stimulated
Voxel #
Voxel #
Voxel #
Voxel #
resting
stimulated
<0.35
0.45
>0.55
Temporal Mutual Information (bits)
D
5mm ROI (Right)
resting
stimulated
Voxel #
Voxel #
Voxel #
Voxel #
resting
stimulated
<0.35
0.45
>0.55
Temporal Mutual Information (bits)

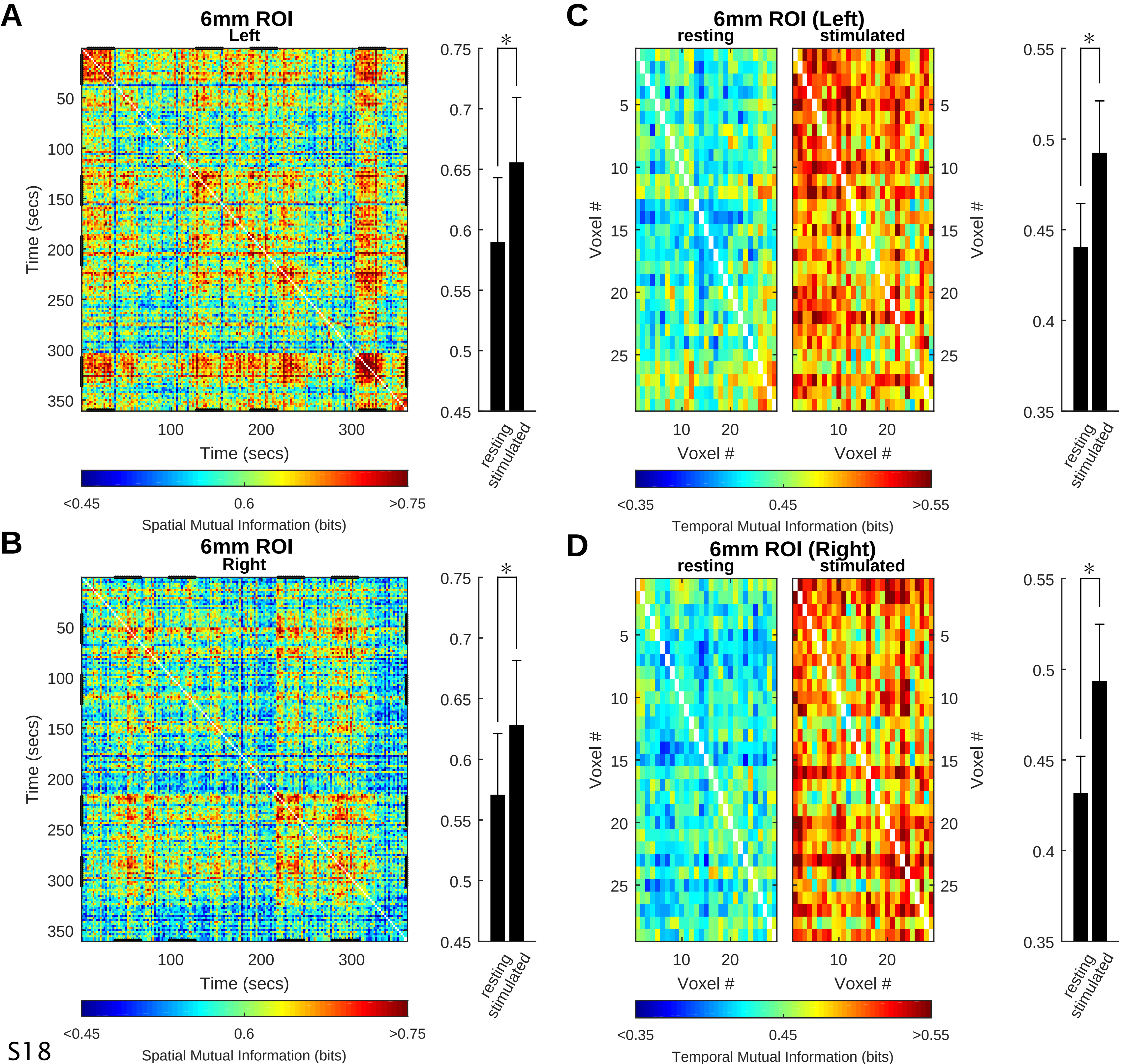
A
6mm ROI
Left
Time (secs)
Time (secs)
Spatial Mutual Information (bits)
<0.45
0.6
>0.75
resting
stimulated
*
B
6mm ROI
Right
Time (secs)
Time (secs)
Spatial Mutual Information (bits)
<0.45
0.6
>0.75
resting
stimulated
*
C
6mm ROI (Left)
resting
stimulated
Voxel #
Voxel #
Temporal Mutual Information (bits)
<0.35
0.45
>0.55
resting
stimulated
*
D
6mm ROI (Right)
resting
stimulated
Voxel #
Voxel #
Temporal Mutual Information (bits)
<0.35
0.45
>0.55
resting
stimulated
*

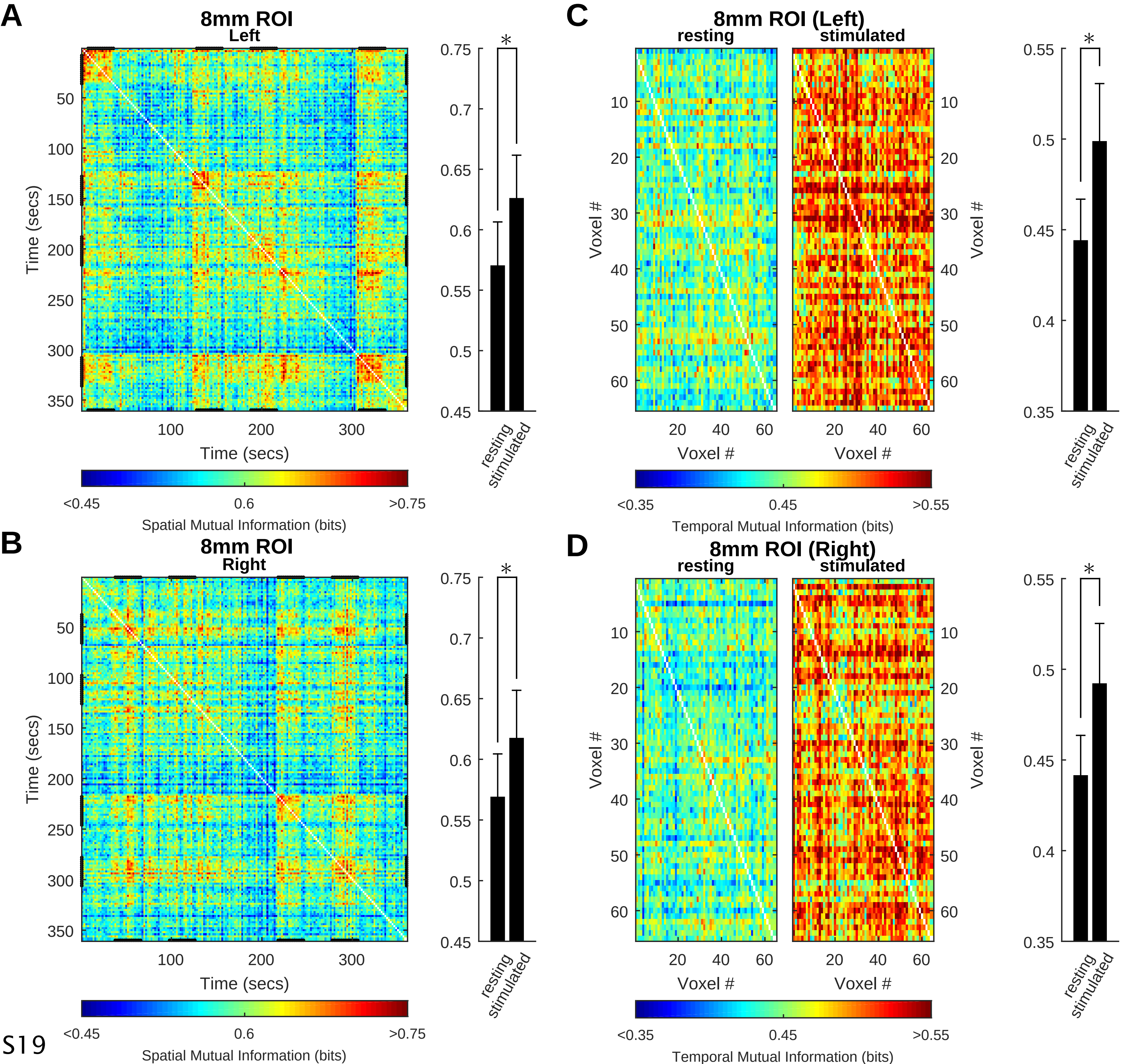

A
8mm ROI
Left
Time (secs)
Time (secs)
resting
stimulated
Spatial Mutual Information (bits)
<0.45
0.6
>0.75
C
8mm ROI (Left)
resting
stimulated
Voxel #
Temporal Mutual Information (bits)
<0.35
0.45
>0.55
B
8mm ROI
Right
D
8mm ROI (Right)

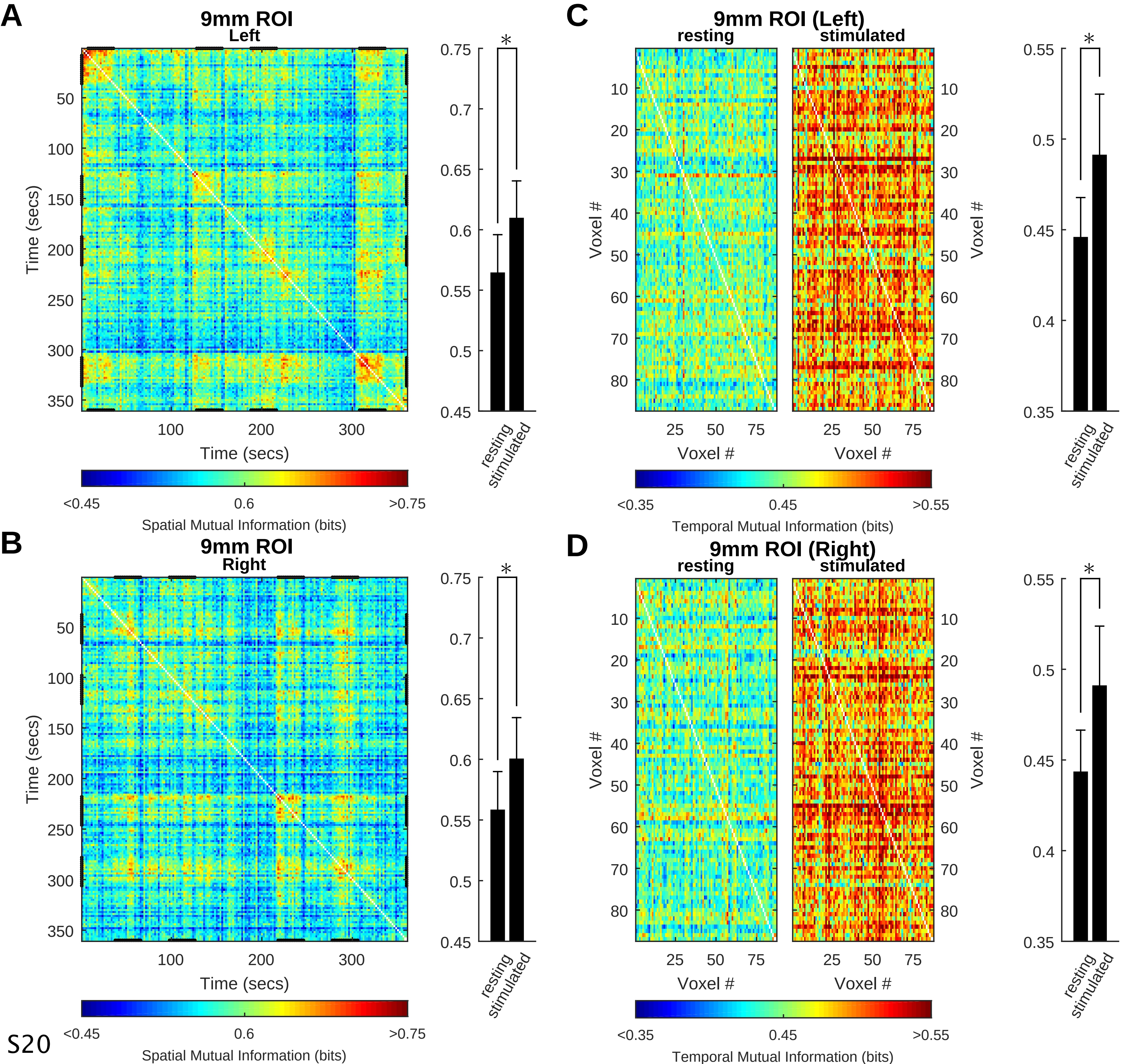
A
9mm ROI
Left
Time (secs)
Spatial Mutual Information (bits)
<0.45
0.6
>0.75
resting
stimulated
*
B
9mm ROI
Right
C
9mm ROI (Left)
resting
stimulated
Voxel #
Temporal Mutual Information (bits)
<0.35
0.45
>0.55
D
9mm ROI (Right)

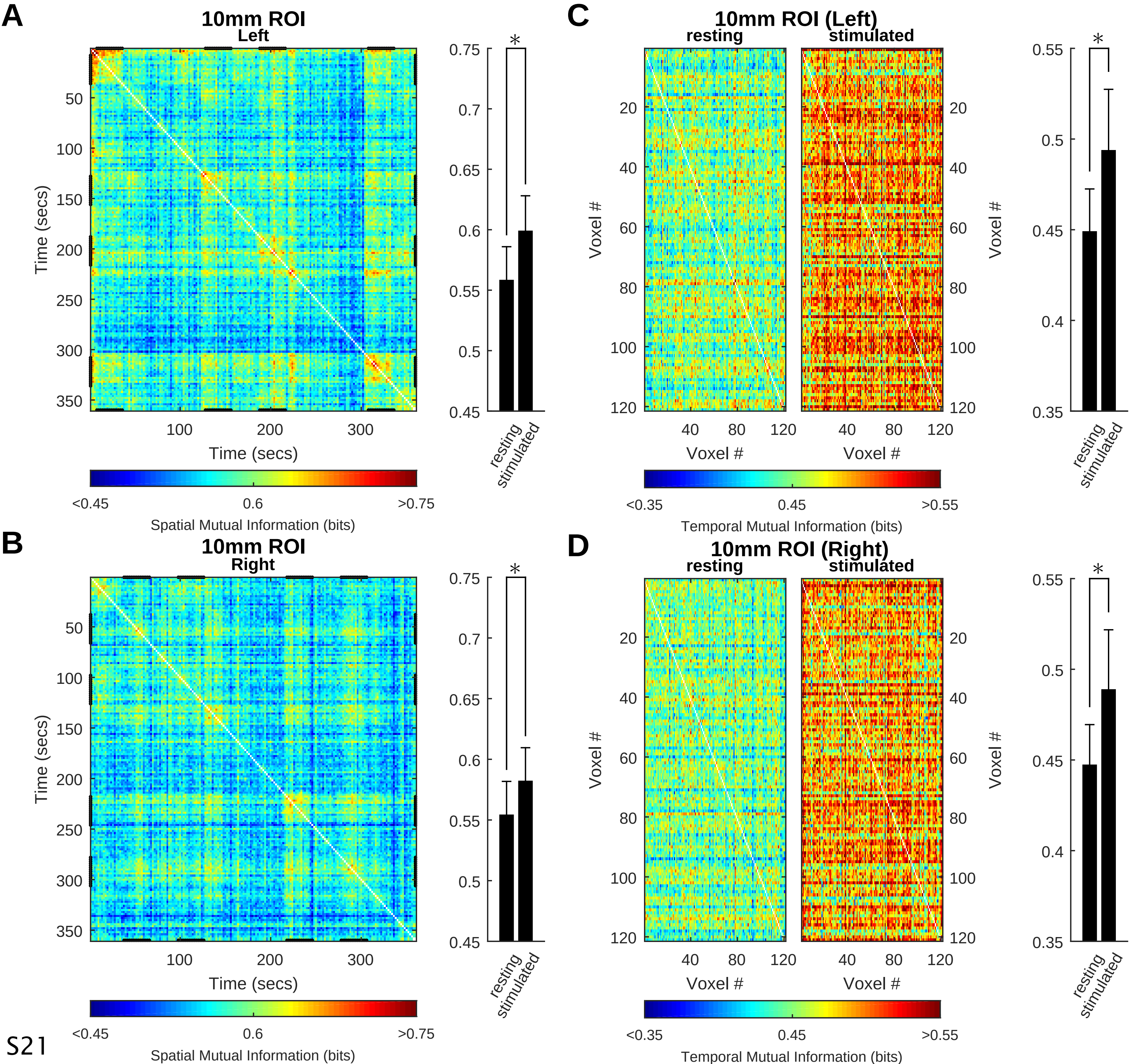
A
10mm ROI
Left
Time (secs)
Time (secs)
Spatial Mutual Information (bits)
<0.45
0.6
>0.75
resting
stimulated
*
B
10mm ROI
Right
Time (secs)
Time (secs)
Spatial Mutual Information (bits)
C
10mm ROI (Left)
resting
stimulated
Voxel #
Temporal Mutual Information (bits)
<0.35
0.45
>0.55
D
10mm ROI (Right)

S21
